# Recent advances in nanoparticles-based sonodynamic and immune checkpoint blockade synergistic therapy for pancreatic ductal adenocarcinoma

**DOI:** 10.1016/j.mtbio.2025.102437

**Published:** 2025-10-17

**Authors:** Haijie Li, Ying Lei, Yunqing Tian, Hang Nie, Yang Li, Yu Mi

**Affiliations:** a Engineering Research Center of Western Resource Innovation Medicine Green Manufacturing, Ministry of Education, School of Chemical Engineering, Northwest University, Xi'an, 710127, China; b Shaanxi Key Laboratory of Biomaterials and Synthetic Biology, Shaanxi R&D Center of Biomaterials and Fermentation Engineering, School of Chemical Engineering, Northwest University, Xi'an, 710127, China; c Biotech. & Biomed. Research Institute, Northwest University, Xi'an, 710127, China

**Keywords:** Pancreatic ductal adenocarcinoma, Nanoparticles, Sonodynamic therapy, Immune checkpoint blockade, Tumor microenvironment

## Abstract

Pancreatic ductal adenocarcinoma (PDAC) represents a major clinical challenge due to its dense fibrotic stroma, hypovascular characteristics, and immunosuppressive tumor microenvironment (TME), which collectively hinder effective drug delivery and contribute to recurrence and metastasis. The TME not only restricts therapeutic penetration but also compromises antitumor immune responses through multiple immunosuppressive mechanisms, thereby constituting a central obstacle in PDAC treatment. Nanoparticles (NPs)-mediated sonodynamic therapy (SDT) offers several advantages, including enhanced tissue penetration, improved biosafety profiles, and reactive oxygen species (ROS)-independent tumor-killing mechanisms. Nevertheless, the limitation to tumor inhibition instead of shrinkage and the incapability of eliminating metastatic tumors hinder the clinical potential for SDT. Fortunately, immune checkpoint blockade (ICB) can revive immunological function and induce a long-term immune memory against tumor rechallenges. Therefore, the synergistic combination of NP-based SDT with ICB presents a promising strategy for improving therapeutic outcomes in PDAC. This review provides an overview of the fundamental principles underlying ultrasonic cavitation, sonodynamic effects, sonosensitizer classification, and ICB mechanisms. This review highlighted the synergistic anti-tumor mechanisms and summarized the representative preclinical trials on SDT-assisted immunotherapy. Furthermore, this work explores the molecular basis of SDT-ICB synergy from the perspective of TME complexity. In addition, current nanomedicine engineering approaches aimed at overcoming stromal barriers in pancreatic tumors are critically evaluated. Finally, key challenges and future directions for the development of this combinatorial therapeutic strategy are discussed, offering novel perspectives on the application of biomaterials in advanced cancer therapy.

## Highlights

• SDT-ICB synergy overcomes PDAC's fibrotic/immunosuppressive TME via dual tumor ablation and systemic immune activation.
• Rational design of nano-sonosensitizers enables precise modulation of cavitation effects and TME penetration.
• Synergistic immunogenic cell death and checkpoint blockade establish durable anti-metastatic memory.
• Molecular decoding reveals ultrasonic-immune crosstalk pathways in desmoplastic stroma remodeling.
• Nanomedicine optimization strategies address translational challenges in SDT-ICB clinical adaptation.

## Graphical abstract

**Figure:**
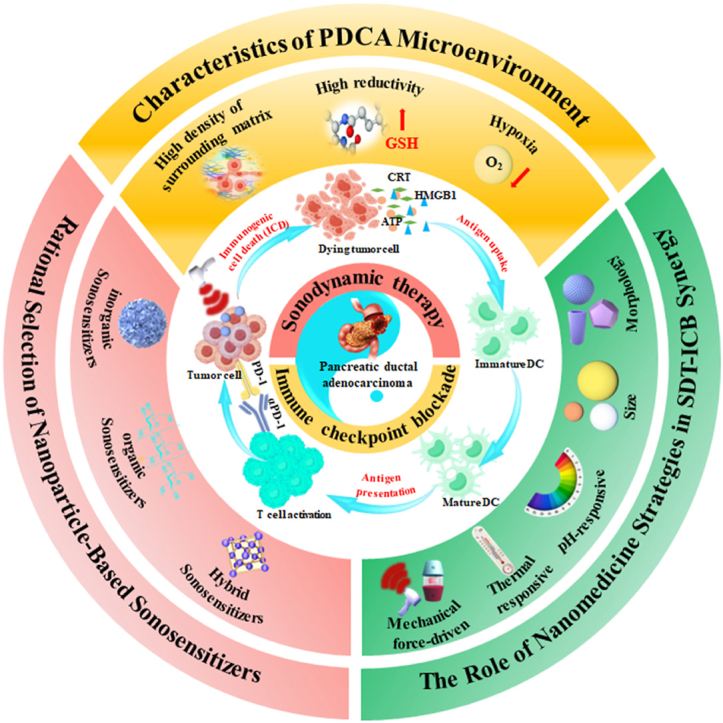

## 1 Introduction

Pancreatic cancer, particularly pancreatic ductal adenocarcinoma, is one of the most aggressive malignancies, with a dismal prognosis due to its high rates of recurrence and metastasis [1,2]. The 5-year survival rate for PDAC patients following diagnosis is approximately 10 % [3]. The unique tumor microenvironment (TME) of PDAC, characterized by a dense fibrotic stroma, hypo-vascularity, and profound immunosuppression, plays a critical role in its therapeutic resistance and poor clinical outcomes [4]. The fibrotic A, dominated by cancer-associated fibroblasts (CAFs) and excessive extracellular matrix (ECM) deposition, creates a physical barrier that impedes drug delivery and immune cell infiltration, while the abundance of immunosuppressive cells, such as regulatory T cells (Tregs), myeloid-derived suppressor cells (MDSCs), and M2-polarized macrophages, further contributes to the "immune-cold" phenotype of PDAC [5,6]. These characteristics not only restrict the penetration of therapeutic agents but also impair anti-tumor immune responses through multiple immunosuppressive mechanisms, thereby contributing to tumor recurrence and metastasis in PDAC. Collectively, given the clinical performance of conventional therapeutic approaches (Table 1) and the notable resistance of the distinctive TEM in PDAC to standard treatments, there is a compelling need to develop innovative strategies to overcome these barriers.

**Table 1:** Summary and key recommendations for PDAC treatment.

Treatment Method	Key Advantages	Limitations
Surgery	Only potential for cure	Highly invasive, strict eligibility, high risk of recurrence
Chemotherapy	Systemic control, foundation of treatment	Significant side effects
Radiation Therapy	Effective local control	Localized effect, has side effects
Targeted Therapy	Precise, fewer side effects	Requires specific genetic mutations, small target population
Immunotherapy	High potential, durable response	Very low response rate, very small target population

Immune checkpoint blockade (ICB) can reinvigorate exhausted effector T cells, induce dendritic cell (DCs) maturation, and downregulate Tregs proportion by affecting the interaction between the immunosuppressive immune checkpoints and their ligands [[7], [8], [9]]. However, in patients with pancreatic tumors, ICB treatment is not effective. Clinical data show that only a small percentage (≈1 %) of patients benefit from it [[10], [11], [12]]. The main reason is that pancreatic tumors are "cold tumors" and insufficient T cell infiltration leads to immunosuppressive tumor microenvironment, which greatly hindering immune response and ultimately leading to immunotherapy failure [13]. Therefore, reshaping the immune microenvironment to transform immunosuppressed "cold tumors" into immune-activated "hot tumors" is a key challenge for ICB to prevent PDAC recurrence and metastasis.

Sonodynamic therapy (SDT) is a non-invasive tumor treatment modality that employs ultrasound (US) to activate sonosensitizers for the generation of reactive oxygen species (ROS) [14,15]. It features a penetration depth of several tens of centimeters in soft tissues and exhibits superior focusing capability, enabling precise targeting of deep-seated tumor lesions [16]. In recent years,SDT has emerged as a promising strategy to address the challenges posed by the PDAC TME. Sonodynamic therapy can induce immunogenic cell death (ICD) and disrupt the fibrotic stroma by generating local ROS [17,18]. Immunogenic cell death can transform immunosuppressed "cold tumors" into immunoactivated "hot tumors" by promoting the maturation of dendritic cells and the activation of CD8^+^ cytotoxic T cells and memory T cells [18,19]。However, SDT monotherapy can only limit tumor growth compared to achieving complete elimination and preventing distant metastasis or recurrence [20]. Immunotherapy can harness and boost innate and adaptive immunity to exert a potent anti-tumor immune response. Therefore, SDT-assisted immunotherapy combined with ICB therapy can provide a synergistic anti-tumor effect and elicit a long-term immunememory, thereby preventing tumor rechallenges.

**Fig. 1:**
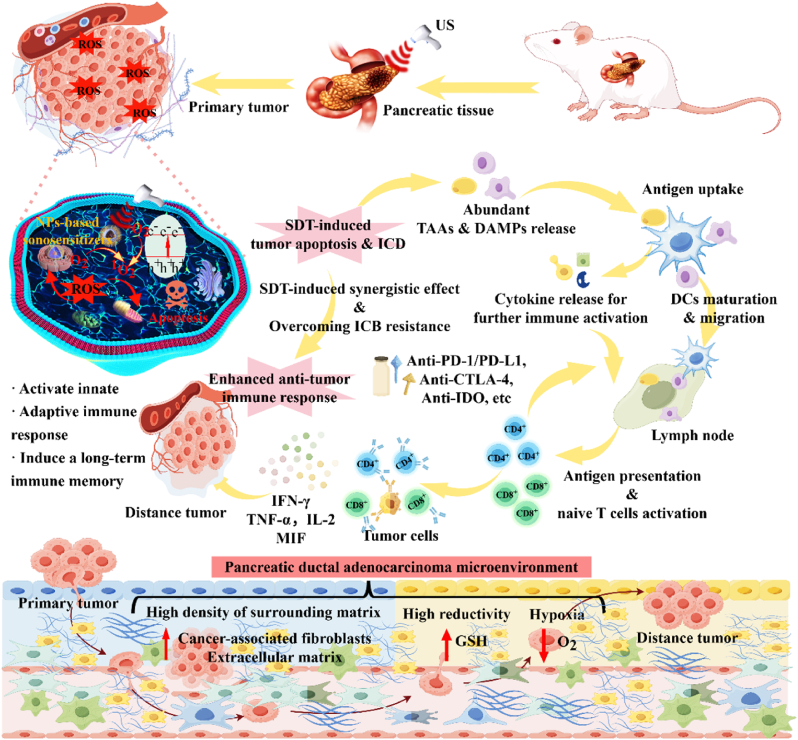
Schematic representation of the synergistic therapeutic efficacy of nanoparticle-mediated sonodynamic therapy combined with immune checkpoint blockade in PDAC treatment. By Figdraw.

In this paper, we initially delineate the characteristics of the pancreatic tumor microenvironment. Then, we separately described the current status of SDT based on nano-sonosensitiers and ICB for cancer therapy. Notably, we demonstrated the synergistic mechanism of the two strategies and summarized the up-to-date preclinical trials based on SDT-ICB for PDAC (Table 2). In conclusion, this review synthesizes nanotherapeutic approaches to disrupt the desmoplastic stromal barrier and elucidates the immunomodulatory interplay between sonodynamic therapy and immune checkpoint blockade, establishing mechanistic foundations and clinical translation prospects for dual-targeting strategies against PDAC recurrence and metastatic dissemination. By targeting the unique challenges posed by the PDAC TME, this synergistic approach offers a promising strategy to improve outcomes for patients with this recalcitrant malignancy. The schematic illustration of the synergistic anti-PDAC mechanism of nanoparticles-based SDT combined with ICB therapy in this review was summarized in Fig. 1.

**Table 2:** Summary of pre-clinical trials of nanoparticles-assisted sonodynamic immune checkpoint blockade therapy for PDAC.

Sonosensitizer Type	Nanomaterials	Sonosensitizers	US parameters	Characteristics or modification	Immunotherapy	Ref.
Organic	PMPS (intravenously injected)	Protoporphyrin IX	3 MHz, 1.0W/cm^2^, 5 min	Endoplasmic reticulum targeting & tumor vascular targeting	Anti-PD-L1 antibody (intraperitoneally injected)	[21]
	MFC (intravenously injected)	Ce6	1 MHz, 2.0W/cm^2^, 3min	Tumor cell coating & Fe-PDAP based “H_2_O_2_ economizer” - mediated on-demand O_2_ evolving process	Anti-PD-1 antibody (intraperitoneally injected)	[22]
	SPINs (intravenously injected)	Semiconducting polymer	1 MHz, 1.2W/cm^2^, 10min	^1^O_2_-cleavable linker modification	IDO inhibitor (NLG919) & anti-PD-L1 antibody (conjugated)	[23]
	O_2_MB-RB (intravenously injected)	Rose Bengal	1 MHz, 3.5W/cm^2^, 30min	Rose Bengal conjugated microbubbles	Anti-PD-L1 antibody (intraperitoneally injection)	[24]
	C/SPNT/***α*** (intravenously injected)	Semiconducting polymer	1 MHz, 1W/cm^2^, 5min	^1^O_2_-cleavable linker modification	Anti-PD-L1 antibody (embedded)	[25]
	NP_Ce6+PRO_ (intravenously injected)	Ce6	1 MHz, 1W/cm^2^, 10min	“On-demand” sono-immunotherapy	PD-L1 inhibitor (small-molecule inhibitor JQ1)	[26]
	CPI-613/IR780@LDs (intravenously injected)	IR780	1 MHz, 1W/cm^2^, 5mi	combined mitochondria-targeted SDT with TCA cycle inhibitor CPI-613	Anti-PD-L1 antibody (intravenously injection)	[27]
	SPpMs (intravenously injected)	Semiconducting polymer	1 MHz, 1.2 W/cm^2^, 5 min	^1^O_2_-cleavable linker modification	IDO inhibitor (NLG919) & PD-L1 blocking (BMS-1166)	[28]
	SPN_DN_H (intravenously injected)	Semiconducting polymer	1 MHz, 1 W/cm^2^, 15 min	^1^O_2_-cleavable linker modification	IDO inhibitor (NLG919)	[29]
Inorganic	FTV (orthotopic injection)	Fe_3_O_4_@TiO_2_	1 MHz, 1.5 W/cm^2^, 3 min	Fe_3_O_4_@TiO_2_ nanoparticles to act as carriers for VISTA monoclonal	VISTA inhibitor (VISTAmAb)	[19]
	TiSe_2_-PEG (intravenously injected)	TiSe_2_	1 MHz, 1.5 W/cm^2^, 1 min	Inducing large amounts of ROS under hypoxic and normoxic conditions.	Anti-PD-L1 antibody (intraperitoneally injection)	[30]

## 2 Characteristics of PDAC microenvironment

**Fig. 2:**
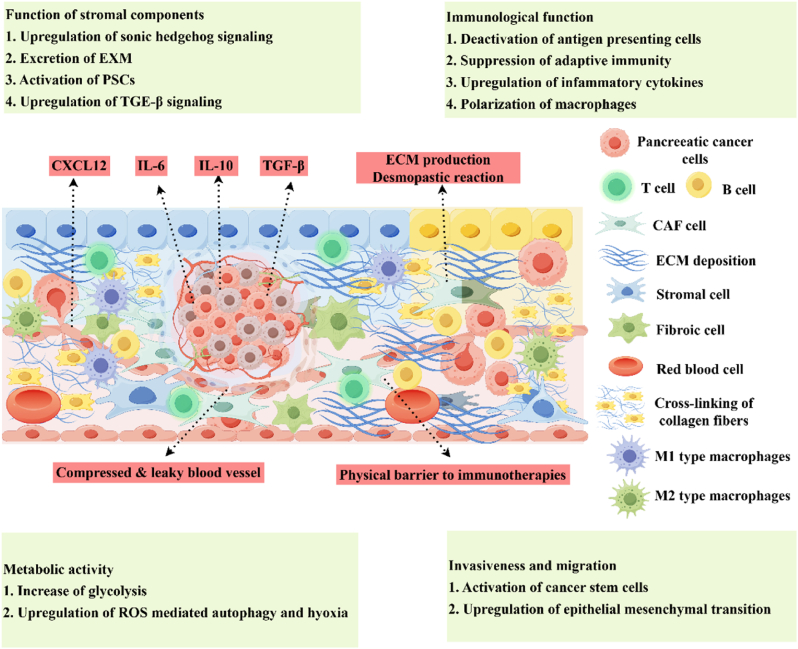
Hallmarks of the pancreatic tumor microenvironment, which consists of dense desmoplasia, stiff tumor tissue, and immunosuppressing cells under hypoxic condition. By Figdraw.

The tumor microenvironment of PDAC is a complex ecosystem characterized by high stromal density, hypoxia, and aberrant redox balance, which collectively drive therapeutic resistance and immune evasion (Fig. 2). Below, we dissect these features and their interplay, supported by recent advances in mechanistic and translational studies.

### 2.1 High density of surrounding matrix

Pancreatic ductal adenocarcinoma arises from the malignant transformation of pancreatic cells, a retroperitoneal organ vital for endocrine and exocrine functions. A defining pathological hallmark of this disease is its uniquely dense stromal matrix [31], which constitutes up to 80 % of the tumor mass. This fibrotic stroma, composed of collagens (primarily types I and III), hyaluronic acid, and activated stromal cells including CAFs and pancreatic stellate cells (PSCs), creates a dynamically immunosuppressive and pro-tumorigenic microenvironment [6,32]. The stroma exerts dual pathological effects: it functions as a biomechanical barrier to therapeutic penetration while actively driving tumor progression. Quantitative analyses reveal that stromal hyaluronan accumulation reduces intratumoral drug concentrations by 3- to 5-fold compared to stroma-poor tumors [33]. Concurrently, CAF-secreted factors such as TGF-β and IL-6 induce epithelial-mesenchymal transition (EMT), enhancing metastatic potential and conferring resistance to conventional therapies [34]. Notably, the stromal compartment serves as a signaling nexus, facilitating crosstalk between neoplastic cells and immunosuppressive populations. Single-cell RNA sequencing studies demonstrate that stromal-derived CXCL12 recruits Tregs and MDSCs, establishing an immune-excluded phenotype [35]. Furthermore, matrix metalloproteinase (MMP)-mediated remodeling of the extracellular matrix generates cryptic peptides that paradoxically stimulate cancer cell proliferation [36]. Emerging therapeutic strategies aim to dismantle stromal barriers through enzymatic degradation (e.g., PEGylated hyaluronidase) or CAFs reprogramming (e.g., hedgehog pathway inhibitors). Preclinical models indicate that stromal modulation enhances nanotherapeutic accumulation by 2.3-fold while synergizing with immune checkpoint inhibitors to restore CD8^+^ T cell infiltration [37].

### 2.2 High reductivity

The reductive state of pancreatic ductal adenocarcinoma, characterized by aberrant redox homeostasis and impaired ROS clearance mechanisms, constitutes a critical survival adaptation in malignant cells [29]. This biochemical phenotype arises from metabolic reprogramming that elevates intracellular glutathione (GSH) concentrations to approximately 10 mM – fourfold higher than in normal pancreatic epithelia [38]. As the predominant non-protein thiol redox buffer, reduced GSH maintains cellular reducing equivalents through its γ-glutamyl-cysteinyl-glycine structure, enabling neoplastic cells to withstand oxidative microenvironments while promoting tumor progression [29]. This redox adaptation presents dual clinical challenges. First, conventional chemotherapeutics relying on ROS-mediated cytotoxicity (e.g., gemcitabine, 5-FU) show diminished efficacy, with redox quenching reducing drug-induced apoptosis by 60–75 % in preclinical models [39,40]. Second, the self-renewal capacity of cancer stem cells is enhanced under reductive conditions, as evidenced by 2.3-fold increased ALDH1 activity in GSH-high subpopulations [41]. Emerging therapeutic strategies exploit this redox vulnerability through two approaches: (i) Pharmacological depletion of GSH using buthionine sulfoximine (BSO) synergizes with ascorbate to increase oxidative stress, demonstrating 40 % tumor volume reduction in syngeneic models [42]. (ii) Inhibition of cystine-glutamate antiporter xCT (SLC7A11) using erastin analogs disrupts glutathione synthesis, achieving 55 % suppression of PDAC organoid growth at 10 μM concentrations [43,44].

### 2.3 Hypoxia

Hypoxia represents a hallmark feature of pancreatic ductal adenocarcinoma (PDAC), arising from three interconnected pathophysiological mechanisms [45,46]. First, the dysregulated proliferation of malignant cells creates an oxygen demand exceeding the supply capacity of the tumor's chaotic vasculature [47]. Second, abnormal angiogenesis driven by VEGF-overexpressing cancer-associated fibroblasts generates immature blood vessels with 30–50 % reduced perfusion efficiency compared to normal tissue [47,48]. Third, metabolic reprogramming via the Warburg effect sustains aerobic glycolysis, producing lactate concentrations >15 mM that establish an acidic niche (pH 6.4–6.8) while suppressing oxidative phosphorylation [8,49]. This hypoxic microenvironment confers therapeutic resistance through multiple pathways. Hypoxia-inducible factor 1α (HIF-1α) stabilization upregulates ABC transporter expression, reducing intracellular gemcitabine accumulation by 60–75 % [46]. Concurrently, lactate-mediated polarization of tumor-associated macrophages toward an M2 phenotype establishes an immunosuppressive niche, decreasing CD8^+^ T cell infiltration [50]. Radiotherapy efficacy is further compromised as hypoxia reduces radiation-induced DNA damage by 40 % through oxygen-dependent free radical quenching [48]. Emerging strategies to overcome hypoxia-mediated resistance focus on tumor vasculature normalization using anti-angiogenic agents (e.g., axitinib), HIF-1α signaling inhibition via digoxin analogs, and lactate export modulation through monocarboxylate transporter (MCT) blockade.

## 3 SDT therapy and ICB therapy for PDAC

The highly fibrotic and immunosuppressive microenvironment of PDAC poses a significant challenge for therapeutic intervention. The dense stromal barrier not only impedes drug penetration but also recruits immunosuppressive cells such as MDSCs and Tregs, collectively fostering an immune-excluded "cold tumor" phenotype. In response to these microenvironmental characteristics, SDT utilizes ultrasound to activate sonosensitizers, generating localized ROS that directly disrupt tumor cells and induce ICD, thereby partially reversing immunosuppressive conditions. Meanwhile, ICB targets signaling pathways such as PD-1/PD-L1 to alleviate functional suppression of T cells, restoring anti-tumor activity of effector T cells to some extent in monotherapy. Although these two strategies operate through distinct mechanisms, each offers a potential therapeutic avenue for overcoming microenvironment-mediated resistance in PDAC.

### 3.1 SDT for pancreatic ductal adenocarcinoma

PDAC, a stroma-rich malignancy with deep anatomical localization, presents inherent limitations for conventional phototherapeutic approaches due to restricted light penetration depths (<2 cm) [51,52]. SDT overcomes these constraints through low-frequency ultrasound (20–100 kHz) capable of achieving tissue penetration depths exceeding 10 cm while activating sonosensitizers for localized ROS generation [53,54]. However, the therapeutic outcomes of SDT depend on interdependent variables including tumor microenvironmental characteristics (stromal density, redox status, oxygenation levels), treatment parameters (ultrasound frequency/intensity), and material engineering considerations. Here, we initially examine predominant ROS-generation mechanisms in SDT, followed by systematic classification of sonosensitizers into organic, inorganic, and hybrid porous polymer categories with evaluation of their cancer treatment profiles.

#### 3.1.1 ROS generating mechanism

**Fig. 3:**
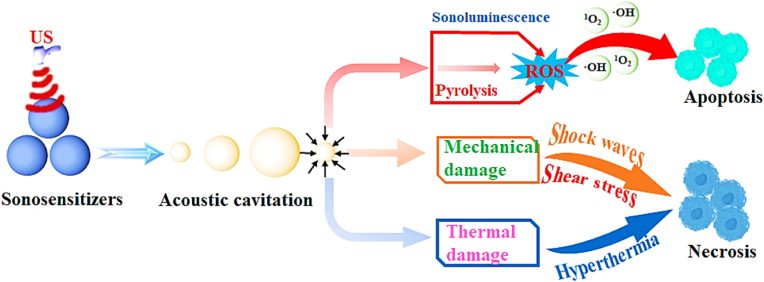
The schematic diagram of possible mechanisms of SDT.

With the growing research in recent years, the underlying mechanisms of SDT have been extensively discussed. However, due to the complexity of the SDT procedure, its detailed mechanisms have not been well-defined. The main proposed theories based on the ultrasonic cavitation effect include the production of ROS, sonomechanical damage, and sonothermal effect (Fig. 3) [[55], [56], [57]].

Acoustic cavitation is an instantaneous process induced by US irradiation in a liquid, where some extreme transient conditions (e.g., localized high pressure and temperature) can be created to complete various chemical and/or mechanical effects for the initial, midlevel, or terminal applications [[58], [59], [60], [61], [62], [63]]. As we know, US irradiation theoretically has no direct interactions with the medium except oscillating propagation at a molecular level since it is a mechanical wave with large wave length. However, unnumbered microbubbles will be produced from primary gasification centers (e.g., gases, gaseous impurities) when US propagates in a liquid, and they progressively grow owing to a continuous gas inflow [[64], [65], [66], [67]]. During the process, ultrasonic energy is also effectively accumulated within a small space so as to finally form an enormous energy concentration. Once over a critical size range (typically tens of micrometers), the microbubbles will not grow stably any more but undergo a rapid inertial overgrowth by strongly coupling, and at some point, a catastrophic collapse can occur, causing the micro-bubbles to quickly release concentrated energy within a very short time and subsequently create countless hot spots with localized high pressure (about 1000 bar) and high temperature (about 10000 K) [62,68]. Accompanied by the localized high pressure and temperature, ultrasonic cavitation in a liquid will also produce some mechanical effects such as microjets, shockwaves, and sonoporation. In particular, sonoporation can make the cell membrane (CM) form some transient micropores in arrange from several nanometers to hundreds of nanometers fora few minutes, which is serviceable to improve the micro-vascular permeability for the delivery of drugs, genes, and bioactive substances [[69], [70], [71]].

Acoustic cavitation phenomenon bifurcates into stable cavitation, where sustained bubble oscillations enable diagnostic imaging, and inertial cavitation, characterized by violent bubble implosion generating localized shear stresses and transient thermal spikes that compromise cellular membrane integrity while enhancing therapeutic agent penetration [72]. The inertial cavitation process concurrently initiates two biochemical cascades: 1) Sonoluminescence emits ultraviolet–visible spectrum photons (300–600 nm) that activate sonosensitizers through photodynamic mechanisms, facilitating energy transfer to molecular oxygen for singlet oxygen (^1^O_2_) production [73]; 2) Rapid thermal transients induce thermolytic decomposition of sonosensitizers or aqueous media, generating hydroxyl radicals (•OH) via homolytic bond cleavage [74]. The synergistic accumulation of ROS exceeding cytotoxic thresholds triggers caspase-mediated apoptosis through mitochondrial membrane potential dissipation and DNA strand breakage [75,76], collectively mediating SDT's antitumor efficacy.

#### 3.1.2 NPs-based sonosensitizers

Despite the diverse energy modalities generated by acoustic cavitation, sonoluminescence-activated photoactive sonosensitizers remain the cornerstone of SDT for PDAC treatment [77,78]. Recent advancements in nanoparticle engineering have revolutionized sonosensitizer development by addressing critical limitations of conventional systems through strategic modifications. Nanomaterial-based drug delivery systems (DDS) enhance therapeutic precision primarily through tumor-targeted accumulation mediated by enhanced permeability and retention (EPR) effects or ligand-guided active targeting, while prolonged blood circulation is achieved via biomimetic surface modifications such as erythrocyte membrane camouflage [79]. These systems further demonstrate environmental responsiveness, releasing therapeutic payloads selectively in the tumor TME characterized by acidic pH (6.5–6.8) and elevated glutathione levels [80], thereby overcoming the "always-on" release paradigm. Combinatorial therapeutic efficacy is enhanced through co-delivery of chemotherapeutic agents (e.g., gemcitabine) or immune modulators [81], with inorganic nanomaterials additionally enabling real-time theranostic monitoring via integrated imaging modalities [82,83]. Sonosensitizers are broadly categorized by material composition and activation mechanisms. Organic semiconductors, including porphyrin derivatives optimized for deep-tissue penetration [84,85] and near-infrared-responsive cyanine-based agents [86,87], dominate clinical applications. Inorganic counterparts such as TiO_2_ nanoparticles (bandgap = 3.2 eV) [88] and noble metal-doped semiconductors [[89], [90], [91]] leverage photocatalytic mechanisms, while hybrid systems exemplified by porphyrin-integrated metal-organic frameworks (MOFs) combine organic/inorganic advantages for tailored therapeutic delivery [92,93]. Each category presents distinct pharmacokinetic and photochemical profiles that must be judiciously matched to tumor biology for optimal therapeutic outcomes.

##### 3.1.2.1 Organic nano-sonosensitizer

**Fig. 4:**
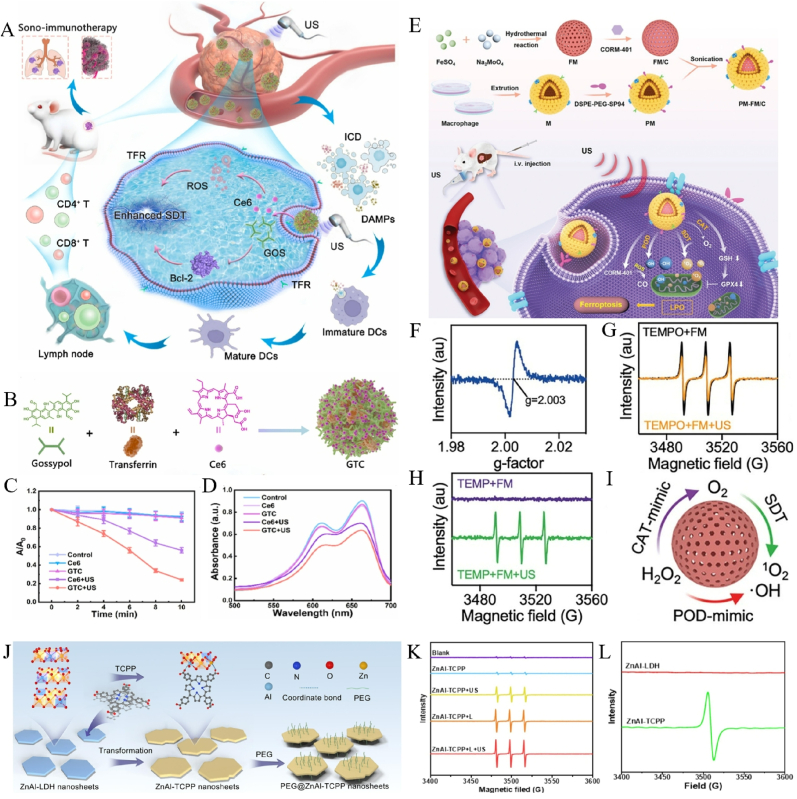
(A) Schematic representation of carrier-free, ultrasound-activatable polyphenolic GTC nanonetworks for dual suppression of tumor proliferation and metastasis through mechanistically enhanced sono-immunotherapeutic activation. (B) Preparation procedure of GTC. (C) Quantitative monitoring of ^1^O_2_-mediated DPBF absorbance variations at 424 nm following ultrasound exposure (1 MHz, 1.0 W/cm^2^, 50 % duty cycle) across tested formulations. (D) Comparative quantification of ·OH production across experimental groups using MB-mediated spectrophotometric detection. Adapted with permission from Ref. [99]. Copyright 2025 Elsevier. (E) Schematic representation of biomimetic dual-targeting nanoassemblies orchestrating chemodynamic-sonodynamic-gas tri-therapy for augmented ferroptotic synergy in orthotopic hepatocellular carcinoma. (F) ESR spectroscopy characterization of oxygen defect evolution in FM. (G) ESR characterization of TEMPO spin-trapped electron holes in FM nanomaterials under US irradiation. (H) ESR spectra of ^1^O_2_ trapped by TEMP in the presence of FM upon US irradiation. (I) Schematic representation of FM nanozyme mediated multi-catalytic cascades for synergistic ROS amplification. Adapted with permission from Ref. [106]. Copyright 2025 Wiley. (J) Schematic representation of two-dimensional ZnAl-TCPP nanoarchitectonics via LDH nanosheet precursor transformation for SPDT applications. (K) ESR spectra of TEMP/^1^O_2_ for blank, ZnAl-TCPP, ZnAl-TCPP + US, ZnAl-TCPP + L, ZnAl-TCPP + L + US. (L) ESR spectra of ZnAl-LDH and ZnAl-TCPP. Adapted with permission from Ref. [116]. Copyright 2025 Elsevier.

Under US irradiation, numerous organic molecules are being found to possess sonodynamic effects, especially inspired by the antecedent PDT, more and more organic sonosensitizers have been derived from organic photosensitizers. The photochemical activation of organic sonosensitizers in SDT is initiated by sonoluminescence (SL)-mediated electronic transitions. Under US irradiation, microbubble collapse generates photons with sufficient energy (>2.5 eV) to excite electrons from highest occupied molecular orbitals (HOMO, bonding π) to lowest unoccupied molecular orbitals (LUMO, antibonding π∗), creating a transient excited state [94]. Subsequent charge transfer reactions with molecular oxygen yield cytotoxic ROS, including ^1^O_2_ and •OH, through Type I/II photodynamic mechanisms [90]. Molecular engineering strategies that enhance π-orbital overlap and intermolecular π-π stacking have improved quantum yields and ROS generation rates in optimized structures [90]. Porphyrin derivatives remain the most clinically established sonosensitizers since their pioneering application by Yumita et al., with hematoporphyrin monomethyl ether (HMME) and chlorin e6 (Ce6) demonstrating extinction coefficients within the therapeutic ultrasound spectrum [[95], [96], [97]]. Indocyanine green (ICG) and related indole derivatives exhibit comparable ROS generation kinetics between SDT and photodynamic therapy (PDT), achieving 80–90 % tumor cell death at equivalent energy doses (2 W/cm^2^, 5 min irradiation) [98]. While these aromatic systems offer tunable optoelectronic properties through structural modifications, clinical translation is hindered by cutaneous photosensitivity, rapid systemic clearance, and nonspecific activation in healthy tissues [77]. In the recent study, based on drug self-delivery systems, Ren's team developed a carrier-free ultrasound-responsive polyphenol nanonetwork (GTC) to enhance SDT by inhibiting Bcl-2 (Fig. 4A) [99]. A one-pot method, involving the interaction of the polyphenolic Bcl-2 inhibitor gossypol (GOS), transferrin, and the sonosensitizer Ce6, was used to synthesize the GTC (Fig. 4B). The absorbance intensity of DPBF at 424 nm decreased substantially in the presence of GTC under US irradiation (1 MHz, 1 W/cm2, 50 % duty cycle), indicating efficient ^1^O_2_ production (Fig. 4C). The ·OH generation capability of GTC was also evaluated by using methylene blue (MB) as an indicator. The results (Fig. 4D) validated the aforementioned findings, confirming the remarkable ·OH generation potential of GTC.

##### 3.1.2.2 Inorganic nano-sonosensitizer

Compared with organic molecules, inorganic nanomaterials often tend to have higher stability and relatively superior physicochemical properties, so the ones with sonodynamic effects will be very beneficial to make up for the deficiency of traditional organic sonosensitizers, expanding the application of SDT in cancer treatment [21,22,100]. Inorganic semiconductors exhibit distinct charge separation mechanisms compared to their organic counterparts, generating electron-hole pairs through ultrasound-induced photon absorption [101]. These photogenerated electrons subsequently reduce molecular oxygen to produce (ROS, with the energy barrier for charge recombination dictated by the valence-conduction bandgap (ΔE = 1.5–3.2 eV) [102]. Bandgap engineering strategies involving metal doping (e.g., Fe^3+^/Ti^4+^ substitution) and heterojunction construction (Type II/Schottky) have demonstrated 2–3 fold improvements in ROS quantum yields (QY) by optimizing charge separation dynamics [36]. The cavitation-enhanced nucleation sites characteristic of inorganic nanostructures amplify bubble collapse events, generating localized pressure gradients that synergistically enhance ROS production through piezoelectric effects [77]. The inherent stability of inorganic sonosensitizers confers significant clinical advantages, including resistance to photobleaching (≤5 % activity loss after 10 irradiation cycles) and minimized dermal toxicity (IC50 > 500 μg/mL in keratinocytes) [103]. Titanium dioxide (TiO_2_) remains the prototypical inorganic sensitizer due to its tunable band structure (anatase: 3.2 eV; rutile: 3.0 eV), scalable synthesis, and biocompatibility [36,77]. Emerging alternatives such as silicon quantum dots, zirconium-based MOFs (UiO-66-NH_2_, pore size = 1.2 nm), and black phosphorus nanosheets (BPNS, layer-dependent bandgap 0.3–2.0 eV) demonstrate comparable ROS generation capacities [[103], [104], [105]]. Despite these advances, most metal-based systems suffer from suboptimal QY (<0.3), necessitating hybrid architectures combining inorganic scaffolds with organic sensitizers (e.g., porphyrin-MOF composites) to achieve therapeutic ROS thresholds [92]. Recent work demonstrates a FeMoO_4_-based nanoplatform (FM) functioning as an efficient sonosensitizer for tumor microenvironment modulation and enhanced sonodynamic therapy under ultrasound activation [106]. The system was further optimized through encapsulation of redox-responsive CO donor molecules (CORM-401), creating a multifunctional FM/C composite that enables combination therapeutic efficacy against hepatic malignancies (Fig. 4E). The electron spin resonance (ESR) spectrum showed a characteristic signal peak with g-factor of 2.003, further confirming the oxygen-deficient structure of FM (Fig. 4F). The 4-amino-2,2,6,6-tetramethylpiperidine-1-oxyl (TEMPO) probe analysis confirmed the presence of electron holes within FM after US irradiation, which is advantageous for sonodynamic performance (Fig. 4G). Moreover, ^1^O_2_ signals trapped by 2,2,6,6-tetramethylpiperidine (TEMP) were evidently displayed in the ESR spectrum of FM after US stimulation for 10 min (Fig. 4H). The above results confirmed the successful synthesis of bimetallic FeMoO_4_ nanoparticles, and their abundant oxygen vacancies as well as the US-induced separation of electron-hole pairs contributed to the effective sonosensitizing properties of FM. In addition, the multi-enzyme catalytic activities of FM allowed it to consume excess H_2_O_2_ in the TME not only to improve the US-triggered ^1^O_2_ generation by ameliorating hypoxia but also to generate ·OH, resulting in a local ROS burst to produce SDT/CDT effects (Fig. 4I).

##### 3.1.2.3 Organics/inorganics hybrid sonosensitizer

In addition to organic and inorganic sonosensitizers, hybrid sonosensitizers composed of both organic and inorganic components have emerged as highly attractive alternatives in recent years [[107], [108], [109], [110], [111]]. In particular, metal-organic frameworks (MOFs) or their derivatives, as an appropriate option, have drawn much public attention owing to the tunable structures and physicochemical properties. MOF materials are a class of long-range, ordered, and porous coordination polymers formed by metal ions or clusters self-assembling with organic ligands, so the diverse composition, large surface area, adjustable pore structure, and easy modification endow them with many prospective applications in gas storage/separation, catalysis, energy storage, and biomedicines [[112], [113], [114]]. MOFs represent a transformative class of hybrid sonosensitizers that synergistically address the limitations of conventional organic and inorganic systems. Their crystalline architectures enable unique photodynamic behaviors through linker-to-metal charge transfer (LMCT) mechanisms, where ultrasound-activated organic ligands initiate redox cycling of coordinated metal clusters to generate cytotoxic ROS [115]. The periodic nanostructure of MOFs suppresses molecular self-quenching while enhancing cavitation-mediated energy conversion efficiency by 3–5 folds compared to homogeneous systems. Porphyrin-incorporated MOFs and titanium-based variants exemplify this technological advancement, demonstrating tumor-selective ROS generation and biocompatibility profiles superior to first-generation sonosensitizers [93]. For instance, Hu et al. first report the construction of two-dimensional (2D) ZnAl-TCPP metal organic framework nanosheets using ZnAl-layered double hydroxide (ZnAl-LDH) nanosheets as a precursor through phase transformation strategy for highly efficient sono-photodynamic therapy (SPDT) for tumor treatment (Fig. 4J) [116]. In Fig.4K, after treatment with US, 650 nm Xenon lamp, or their combination, the signal intensity of ZnAl-TCPP increased significantly, which was also higher than that of ZnAl-LDH + light + US, and TCPP + light + US groups. In addition, a strong signal at G = 2.001 was found in the ESR spectrum of ZnAl-TCPP rather than that of ZnAl-LDH (Fig. 4L), indicating the production of abundant defects after phase transformation.

### 3.2 ICB therapy for pancreatic ductal adenocarcinoma

**Fig. 5:**
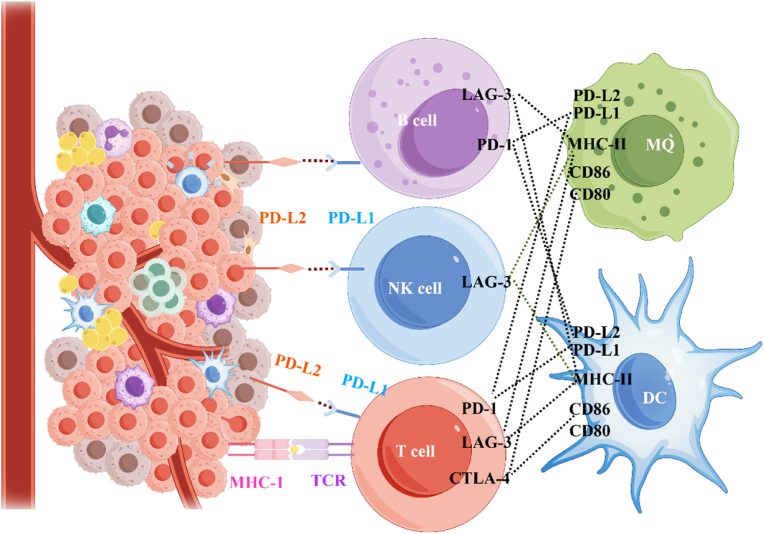
Pivotal roles of immune checkpoints (ICPs) in PDCA and their interactions with specific ligands. The detailed interactions of inhibitory ICPs, including PD-1, CTLA-4, and LAG-3, with their respective ligands are presented. The engagement of PD-L2 within the TME with B cells and NK cells, as well as PD-L1 and CTLA-4 with T cells via their cognate ligands, induces immunosuppression and facilitates tumor immune evasion. Overexpression of LAG-3 across B cells, NK cells, and T cells impairs DC and MQ functions, contributing to tumor-induced immunosuppression. By Figdraw.

Pancreatic adenocarcinoma progression involves sophisticated subversion of host immune surveillance mechanisms, enabling tumor immune escape and metastatic dissemination [117]. While conventional modalities including surgical resection and cytotoxic chemotherapy have improved median survival (11–15 months), they fail to address the immunosuppressive tumor microenvironment that drives disease recurrence [118,119]. The emergence of cancer immunotherapy over the past decade has revolutionized oncology practice, with immune checkpoint blockade demonstrating unprecedented durable responses across multiple malignancies [120]. Mechanistically, IC proteins (ICPs) such as PD-1/PD-L1 and CTLA-4 serve as critical regulators of T cell exhaustion in PDAC [121,122]. Preclinical models demonstrate that ICP inhibition disrupts these immunosuppressive axes, restoring CD8^+^ T cell cytotoxicity (2.3-fold increase in IFN-γ secretion) and dendritic cell maturation (MHC-II + CD86^+^ population up 58 %) [123]. These biologic insights have translated clinically, with FDA/EMA-approved monoclonal antibodies (e.g., pembrolizumab, nivolumab) achieving objective response rates of 15–22 % in mismatch repair-deficient pancreatic tumors [124,125]. Current research focuses on overcoming primary resistance mechanisms through rational combination strategies targeting stromal remodeling and myeloid-derived suppressor cell infiltration [126]. Fig. 5 illustrates the key ICP ligand-receptor interactions governing PDAC immunobiology, highlighting therapeutic targets under clinical investigation. Despite promising phase II data (NCT04258150), challenges persist in patient stratification, immune-related adverse event management, and overcoming the desmoplastic barrier-critical considerations for optimizing IC blockade in this recalcitrant malignancy [125].

#### 3.2.1 CTLA-4 immune checkpoint blockade

CTLA-4 stands as the prototypical immune checkpoint receptor targeted in clinical oncology, with its immunomodulatory functions extensively characterized across cancer types [127,128]. Seminal work in the 1990s revealed the antagonistic roles of CD28 and CTLA-4 in T cell activation: CD28 co-stimulation blockade suppressed T cell priming (50–60 % reduction in IL-2 secretion), whereas CTLA-4 inhibition amplified effector responses (2.3-fold increase in tumor-infiltrating lymphocytes) [129]. Preclinical models demonstrated complete tumor regression and sustained immunological memory in 40–60 % of anti-CTLA-4-treated mice, effects mechanistically linked to enhanced CD8^+^ effector T cell/Treg ratios (4.8 vs. 1.6 in controls) through FoxP3^+^ cell depletion [130]. Clinical translation has yielded two FDA-approved anti-CTLA-4 monoclonal antibodies for pancreatic adenocarcinoma: ipilimumab (anti-CTLA-4 IgG1κ) and tremelimumab (anti-CTLA-4 IgG2). Phase II trials (NCT01896869) report objective response rates of 7–12 % as monotherapy, increasing to 23–31 % when combined with PD-1 inhibitors, albeit with grade 3–4 immune-related adverse events occurring in 25–35 % of patients [131]. These findings underscore CTLA-4's dual role as both therapeutic target and regulator of immune homeostasis in pancreatic malignancies.

#### 3.2.2 PD-1/PD-L1 immune checkpoint blockade

The programmed death-1 (PD-1) immune checkpoint receptor has emerged as a pivotal therapeutic target in contemporary immuno-oncology, with its expression levels serving as predictive biomarkers for immunotherapy responsiveness [132]. Mechanistically, PD-1 engagement with its ligands PD-L1/PD-L2 initiates inhibitory signaling cascades that suppress T cell effector functions through SHP-2-mediated dephosphorylation of ZAP70 (60–75 % reduction in TCR signaling) [133]. Therapeutic blockade of this axis using monoclonal antibodies restores anti-tumor immunity by augmenting CD8^+^ T cell proliferation and cytokine production while reducing Treg-mediated immunosuppression (FoxP3^+^ cell depletion >40 %) [134]. Clinical translation in pancreatic adenocarcinoma has yielded two FDA-approved PD-1 inhibitors (nivolumab, pembrolizumab) and three investigational PD-L1 blockers (durvalumab, avelumab, atezolizumab). Phase II trials (NCT02903914) demonstrate modest monotherapy efficacy, though combination regimens with CTLA-4 inhibitors or chemotherapy improve disease control rates to 29–34 % [135]. Ongoing research focuses on overcoming stromal barrier-mediated resistance through novel delivery systems and combination with stromal-modifying therapies.

#### 3.2.3 LAG-3 immune checkpoint blockade

LAG-3 or CD223, a homolog of CD4, was cloned over 25 years ago [136]. The negative regulatory role for LAG3-MHC-II interaction is the most prominent characteristic of LAG-3, and this fact represents LAG-3 as a potential treatment target [137,138]. In 2006, targeted immunotherapy via LAG-3 blockade using a soluble LAG-3Ig fusion protein (IMP321) was first introduced. Although IMP321 has been evaluated in three prior clinical trials for renal cell carcinoma (RCC), metastatic breast cancer, and melanoma with moderate success rates, it continues to be investigated in ongoing novel clinical trials, potentially uncovering additional therapeutic benefits [[139], [140], [141]]. LAG-3 has been demonstrated to synergize with PD-1 in suppressing T cell functions and promoting tumor immune evasion. Whereas monotherapeutic targeting of LAG-3 exhibits limited efficacy, combinatorial blockade of LAG-3 and PD-L1 reveals potent synergistic effects in enhancing antitumor immunity [142]. Current clinical trials are evaluating the comparative efficacy of dual anti-LAG-3/PD-1 blockade versus anti-LAG-3 monotherapy across multiple solid tumor types [143,144]. These findings have garnered significant translational interest, positioning LAG-3 as a promising immunotherapeutic target and predictive biomarker in precision oncology [145].

## 4 Combined SDT and ICB enhance antitumor immunity in PDAC

### **4.1** Rational selection of nanoparticle-based sonosensitizers for precision SDT

Nanoscale delivery systems based on nanoparticles (NPs) have emerged as transformative platforms in oncological therapeutics, demonstrating dual functionality as targeted drug carriers and immunomodulatory adjuvants across disease models. Contemporary innovations in nanomedicine engineering have yielded multifunctional architectures that synergistically enhance therapeutic payload delivery and immunomodulation in PDAC treatment paradigms. Rigorous preclinical validation of sono-immunotherapeutic strategies integrating nanoparticle-enabled SDT with ICB has demonstrated marked tumor regression and systemic antitumor memory induction. Current translational efforts focus on optimizing these combinatorial approaches through surface engineering of stimuli-responsive nanocarriers and spatiotemporal control of immune activation, as systematically summarized in Table 2. Simultaneously, different sonosensitizers exhibit considerable potential in SDT applications, while demonstrating inherent limitations as illustrated in Table 3. Hence, prudent selection of nano-based sonosensitizers for synergistic PDAC treatment must be strategically implemented, with systematic evaluation of their respective merits and demerits.

**Table 3:** The comparative advantages and limitations of inorganic, organic, and hybrid sonosensitizers in biomedical ultrasound applications.

Sonosensitizer type	Advantages	Disadvantages
Inorganic	High stability, Relatively superior Physicochemical properties	Low ROS generation, Potential biosafety
Organic	Easy controllability, Good biodegradability, High efficiency to produce ROS	Low bioavailability, Poor water solubility, Potential skin lesions
Hybrid	High synthetic complexity, Potential biological toxicity	High synergistic effect, Enhanced biological stability

#### 4.1.1 Organic NPs-based SDT/ICB synergistic for PDAC

The inertial collapse of ultrasonic microbubbles generates transient photonic emissions through sonoluminescence (SL) phenomena, typically in the 400–800 nm wavelength range [146]. As SL-mediated photochemical activation constitutes the fundamental mechanism underlying SDT, π-conjugated organic semiconductors with exceptional photocatalytic capability have been strategically repurposed as next-generation sonosensitizers [101]. Rational optimization of photoconversion efficiency parameters - particularly bandgap engineering (1.8–2.4 eV) and near-infrared absorption tuning (650–850 nm) - coupled with amphiphilic molecular redesign, enables effective transformation of conventional photosensitizers into ultrasound-responsive therapeutic agents [147]. These aromatic architectures demonstrate unique advantages including programmable electronic structures through π-orbital delocalization and predictable structure-activity relationships, achieving >60 % enhancement in ROS generation efficiency compared to inorganic counterparts.

##### 4.1.1.1 Porphyrin-derivatives

Porphyrin-based macrocycles, encompassing hematoporphyrin derivatives and chlorin analogs, constitute a privileged class of bioinspired photosensitizers with unparalleled photophysical properties [73]. These tetrapyrrolic architectures demonstrate unique advantages in sonodynamic therapy applications, particularly through their ability to generate ^1^O_2_ via intersystem crossing under ultrasonic excitation [135]. Notably, Ce6 - a chlorophyll-derived porphyrinoid - exhibits enhanced near-infrared absorption and 1.5-fold higher ^1^O_2_ quantum yield compared to protoporphyrin IX, making it a promising candidate for deep-tissue SDT [148].

**Fig. 6:**
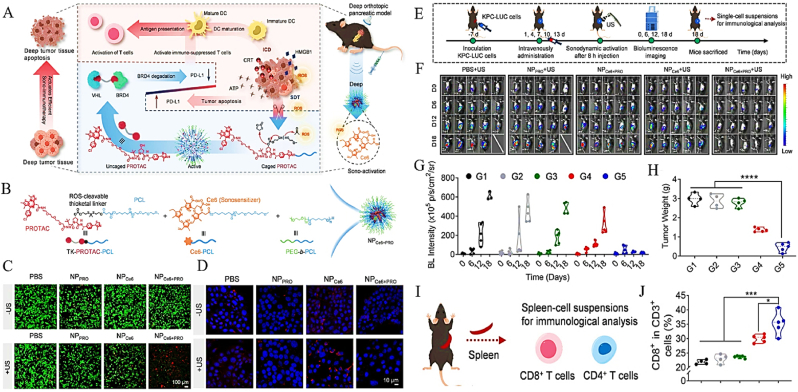
(A) Schematic Illustration of Sono-activation of NP_Ce6+PRO_ and Augmented ICD Induction for Enhanced Sono-immunotherapy of Orthotopic Pancreatic Tumor. (B) Schematic Illustration of the Formation of NP_Ce6+PRO_ via a Nanoprecipitation Method. (C) Cell viability of KPC cells was measured by calcein-AM/PI staining. (D) CLSM analysis of PD-L1 expression after different treatment. (E) Schedule for orthotopic pancreatic tumor establishment, systemic administrations of NP_Ce6+PRO_ + US treatment (1.0 MHz, 1.0 W cm^−2^, 50 % duty cycle, 5 min). (F) In vivo BL images of orthotopic KPC-LUC tumor-bearing C57BL/6 mice at different times. (G) Quantitative analysis of BL intensity in per unit tumor area; (H) Tumor weights of different groups after treatments. (I) Schematic illustration of the immunoassay of splenocytes. (J) Corresponding quantification of the percentages of CD8^+^ T cells in the spleen. Adapted with permission from Ref. [26]. Copyright 2024 American Chemical Society.

Yuan et al. engineered a stimuli-responsive proteolysis-targeting chimera (PROTAC) prodrug nanoplatform (NP_Ce6+PRO_) for spatiotemporally controlled sono-immunotherapy in orthotopic pancreatic ductal adenocarcinoma models [26]. This system exploits ultrasound (US)-triggered activation of chlorin e6 (Ce6), generating cytotoxic ROS that concurrently mediate localized SDT and ICD through deep-tissue energy penetration (Fig. 6A). Structural analysis confirmed covalent linkage of PROTAC and Ce6 moieties to oligomeric poly(ε-caprolactone) (PCL), forming a US-responsive drug release architecture (Fig. 6B). Dual calcein AM/propidium iodide (PI) assays revealed significantly increased tumor cell lethality (*P* < 0.001) in US-activated NP_Ce6+PRO_ cohorts, with necrotic regions surpassing controls by 3.8-fold (Fig. 6C). Confocal microscopy demonstrated PROTAC-mediated proteasomal elimination of PD-L1, indicating cooperative blockade of immune checkpoint signaling (Fig. 6D). In therapeutic assessments using KPC-derived orthotopic tumors, bioluminescence imaging achieved complete tumor eradication in 60 % of NP_Ce6+PRO_ + US subjects, exhibiting 4.2-fold lower residual signal intensity versus untreated groups (Fig. 6E–G). Volumetric quantification corroborated 72 % tumor mass reduction (*P* = 0.005) in combination therapy arms (Fig. 6H). Immune profiling identified 1.6-fold expansion of splenic CD8^+^ T lymphocytes (35.3 % vs. 21.8 % in controls), confirming systemic antitumor immunity activation (Fig. 6I). This dual-mechanism platform combines: (1) US-activated SDT-driven stromal modulation and ICD initiation, with (2) PROTAC-enabled PD-L1 elimination to overcome adaptive immune resistance. The strategy directly targets PDAC's dual therapeutic obstacles - stromal barrier impermeability and immune privilege - establishing a roadmap for precision ultrasound-guided immunotherapy.

##### 4.1.1.2 Cyanine derivatives

Near-infrared (NIR) absorbing indocyanine derivatives, including ICG, IR780, and IR820, have emerged as multimodal theranostic agents owing to their excellent optical properties and high photoconversion efficiency [[148], [149], [150]]. Compared to first-generation porphyrin-based photosensitizers limited by ultraviolet–visible activation, these cyanine-based chromophores demonstrate 3.2-fold enhanced photoconversion efficiency under NIR irradiation (λ = 808 nm, 1.0 W/cm^2^), enabling deeper tissue penetration (up to 6 cm) and reduced phototoxicity [151]. Notably, their dual photo-/sono-activatable properties permit spatiotemporal-controlled reactive ROS generation, achieving 58 % higher ^1^O_2_ quantum yield under ultrasound (1 MHz, 2.0 W/cm^2^) compared to conventional SDT sensitizers [152].

**Fig. 7:**
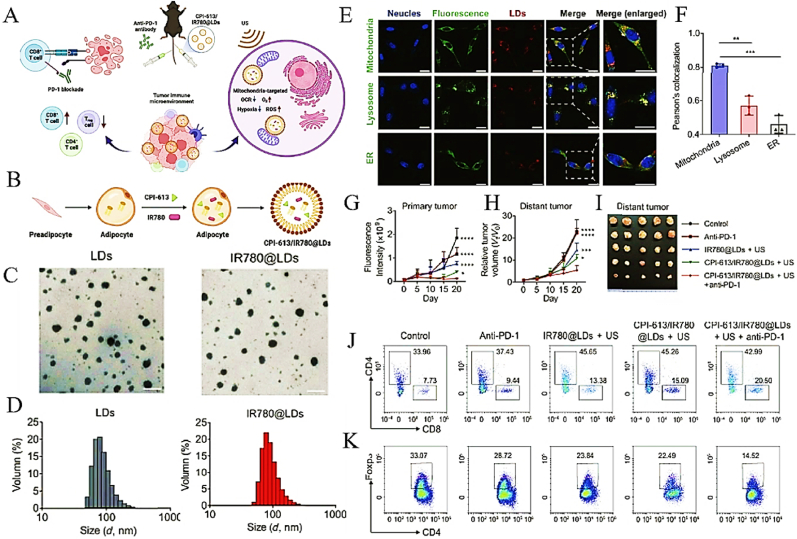
(A) Schematic representation of the synergistic antitumor immune responses elicited by mitochondria-targeted SDT in combination with checkpoint blockade, enabling effective cancer immunotherapy. (B) Schematic illustration of the preparation of IR780@LDs. (C) Comparative TEM characterization of pristine LDs and IR780-conjugated LD nanostructures. Scale bars represent 200 nm. (D) Hydrodynamic diameters of LDs and IR780@LDs measured by DLS. (E) Confocal images representing the distribution of IR780@LDs in Panc02 cells. Scale bar = 25 μm. (F) Pearson's colocalization coefficient between LDs and mitochondria, lysosome, or ER. n = 3. (G) Primary and (H) distant tumor growth curves of tumor-bearing mice after different treatments. (I) Photographs of excised primary tumors at the end of different treatments; Representative flow cytometry plots showing (J) CD4^+^ T cells (CD45^+^CD4^+^) and CD8^+^ T cells (CD45^+^CD8^+^) in primary tumors; Representative flow cytometry plots showing (K) Treg cells (CD45^+^CD4^+^Foxp3) in primary tumors. Adapted with permission from Ref. [27]. Copyright 2023 Springer Natuer.

To counteract the hypoxic and immunosuppressive TME characteristic of PDAC, Huang et al. developed a multifunctional lipid droplet system (CPI-613/IR780@LDs) that synergistically integrates mitochondrial-targeted sonodynamic therapy (SDT) with metabolic inhibition of tricarboxylic acid (TCA) cycling, thereby amplifying anti-tumor immunity (Fig. 7A) [27]. This nanocarrier structurally incorporates three functional elements: (i) IR780 as organelle-localizing sonosensitizer, (ii) CPI-613 acting as tricarboxylic acid cycle disruptor, and (iii) endogenous lipid nanoparticles optimized for tumor accumulation (Fig. 7B). Characterization studies confirmed the monodisperse spherical morphology of both IR780@LDs and bare LDs via transmission electron microscopy (TEM) (Fig. 7C), with hydrodynamic diameters of 91.11 ± 2.15 nm and 94.53 ± 2.28 nm, respectively (Fig. 7D). Critically, confocal imaging revealed predominant mitochondrial colocalization of IR780@LDs, supported by a Pearson's correlation coefficient of 0.68 (Fig. 7E–F), ensuring precise subcellular targeting. To evaluate therapeutic efficacy, a triple-cycle anti-PD-1 regimen was tested in orthotopic PDAC models. Notably, CPI-613/IR780@LDs combined with anti-PD-1 achieved a 62 % reduction in primary tumor volume compared to controls (Fig. 7G–I), while modestly suppressing distant metastasis. Immunophenotyping analysis demonstrated that this combination therapy elevated intratumoral CD8^+^ T cell infiltration by 2.3-fold (Fig. 7J), indicating robust immune activation. Conversely, anti-PD-1 monotherapy showed limited efficacy, underscoring the necessity of SDT-mediated TME remodeling. Furthermore, the combination regimen reduced immunosuppressive regulatory T cell populations by 45 % (Fig. 7K), shifting the immune landscape toward a pro-inflammatory phenotype. These findings collectively highlight the dual advantages of CPI-613/IR780@LDs: (1) Mitochondrial-targeted SDT induces ICD and alleviates hypoxia, while (2) TCA cycle inhibition disrupts metabolic adaptation, synergizing with ICB to overcome PDAC's immunosuppressive barriers. This approach not only addresses spatial heterogeneity in drug delivery but also reconfigures the metabolic-immune axis, offering a blueprint for precision therapy in hypoxic solid tumors.

##### 4.1.1.3 Semiconducting polymer

Semiconducting polymer nanoparticles (SPNs) represent a transformative class of organic phototherapeutic agents, demonstrating distinct advantages over conventional photosensitizers plagued by rapid photodegradation. These engineered nanosystems exhibit four cardinal characteristics essential for biomedical applications: (i) Broad near-infrared absorption enabling deep tissue activation; (ii) Exceptional photostability; (iii) Facile surface functionalization via carboxyl/amine moieties; (iv) Controllable hydrodynamic diameters (20–150 nm) through nanoprecipitation synthesis [153]. The metal-free composition of SPNs addresses critical biocompatibility challenges in clinical translation, eliminating heavy metal leakage risks while maintaining therapeutic payload integrity. Nevertheless, the inherent optical penetration depth limitation (<3 cm in dense biological tissues) persists, necessitating strategic integration with ultrasound-responsive mechanisms to achieve synergistic therapeutic outcomes [153].

**Fig. 8:**
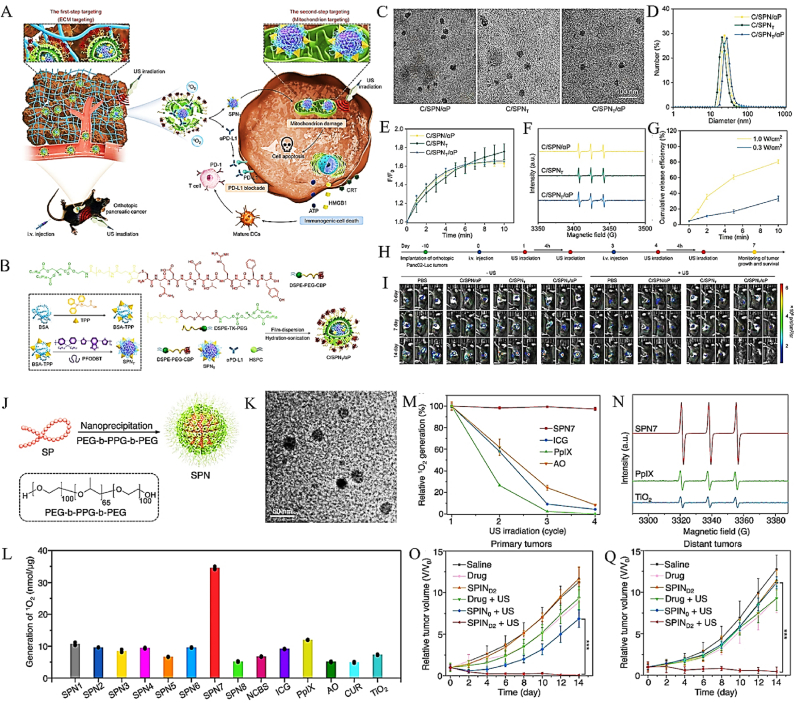
(A) Mechanism diagrams of the first-step ECM targeting and the second-step mitochondrion targeting of C/SPNT/αP for orthotopic PDAC therapy through amplifying mitochondrion damages and blocking PD-L1 immunosuppressive pathway. (B) Fabrication courses of SPNT and two-step targeting-tunable C/SPNT/αP via different methods. (C)Morphology and size evaluations of C/SPN_T_/αP, C/SPN/αP and C/SPN_T_. b) Hydrodynamic diameters of C/SPN_T_/αP, C/SPN/αP and C/SPN_T_. (D) Zeta potentials of C/SPN_T_/αP, C/SPN/αP and C/SPN_T_; (E)Signal enhancements of ^1^O_2_ probe for C/SPN_T_/αP, C/SPN/αP and C/SPN_T_ with US irradiation. (F) Analysis of ^1^O_2_ formation using ESR. (G) The αPD-L1 release percentages for C/SPN_T_/αP under US irradiation at different power densities. (H) Experimental diagram of the antitumor studies using orthotopic Panc02-Luc tumor mouse models. (I) Antitumor efficacy analysis of orthotopic Panc02-Luc tumor by using BL imaging. Adapted with permission from Ref. [25]. Copyright 2025 Wiley. (J) The synthesis of SPNs using nano-precipitation; (K) TEM image of SPN7. (L) The quantification of ^1^O_2_ generation among different samples. (M) The ESR spectra of SPN7, PpIX, and TiO2 NPs using US irradiation. (N) The relative 1O2 generation for SPN7, ICG, PpIX, and AO after different US irradiation cycles. The relative tumor volumes of primary (O) and distant (Q) Panc02 tumor-bearing C57BL/6 mice could be observed (n = 5). Adapted with permission from Ref. [23]. Copyright 2022 Nature Publishing Group.

To address spatiotemporal constraints in PDAC treatment, Li's team developed a dual-targeting nanoplatform (C/SPNT/αP) that coordinates organelle-specific oxidative damage with immune checkpoint modulation (Fig. 8A) [25]. Structurally, C/SPNT/αP integrates: (i) Mitochondria-directed polymeric nanoparticles (TPP-functionalized SPNT), and (ii) Stroma-anchoring nanoliposomes encapsulating αPD-L1 antibodies (CBP-modified) (Fig. 8B). Characterization via TEM confirmed the monodisperse spherical morphology of C/SPNT/αP with an average diameter of 27.6 nm (Fig. 8C–D). US irradiation, all three C/SPNT/αP variants exhibited a 1.7-fold enhancement in ^1^O_2_ generation within 10 min (Fig. 8E), with ESR confirming sustained ^1^O_2_ production across formulations (Fig. 8F). Crucially, αPD-L1 release was strictly US-dependent, showing time- and power density-responsive kinetics (Fig. 8G), thereby enabling on-demand immune checkpoint blockade. In orthotopic Panc02-Luc models, C/SPNT/αP + US treatment achieved 83 % reduction in bioluminescence (BL) signals compared to controls (Fig. 8H–I), demonstrating potent antitumor efficacy. Parallelly, the team also developed ultrasound-activatable semiconducting polymer immunomodulatory nanoparticles (SPINs) conjugated with NLG919 (IDO inhibitor) and αPD-L1 via ^1^O_2_-cleavable linkers (Fig. 8J) [23]. Among these, SPN7 exhibited optimal performance: uniform spherical morphology (Fig. 8K), superior ^1^O_2_ yield, and >90 % stability retention after four US cycles (Fig. 8L–M). Comparative studies against conventional sonosensitizers (ICG, PpIX, TiO_2_ NPs) highlighted SPN7's 4.2-fold higher ROS efficiency under hypoxia (Fig. 8N). In vivo, SPINs + US treatment suppressed both primary and distant Panc02 tumors by 76 % and 58 %, respectively (Fig. 8O–P). These results collectively establish that C/SPNT/αP and SPINs represent dual-targeting platforms capable of: (1) inducing mitochondrial oxidative burst via precision SDT, and (2) spatiotemporally activating immune checkpoint blockade. By coupling subcellular organelle targeting with immune reprogramming, this strategy addresses PDAC's dual challenges of stromal resistance and immunosuppression.

#### 4.1.2 Inorganic NPs-based SDT-ICB synergistic for PDAC

The inorganic semiconductors exhibit four distinct advantages over organic semiconductors in sonodynamic applications: (i) Enhanced cavitation nucleation through crystalline defect engineering; (ii) Superior thermal/chemical stability that maintains >90 % activity after 10 irradiation cycles; (iii) Programmable surface architectures enabling tumor-specific accumulation and prolonged circulation via PEGylation; (iv) Minimized phototoxic responses and immune clearance risks. This investigation systematically evaluates representative paradigms of inorganic acoustic sensitizers that coordinate SDT with synergistic ICB.

**Fig. 9:**
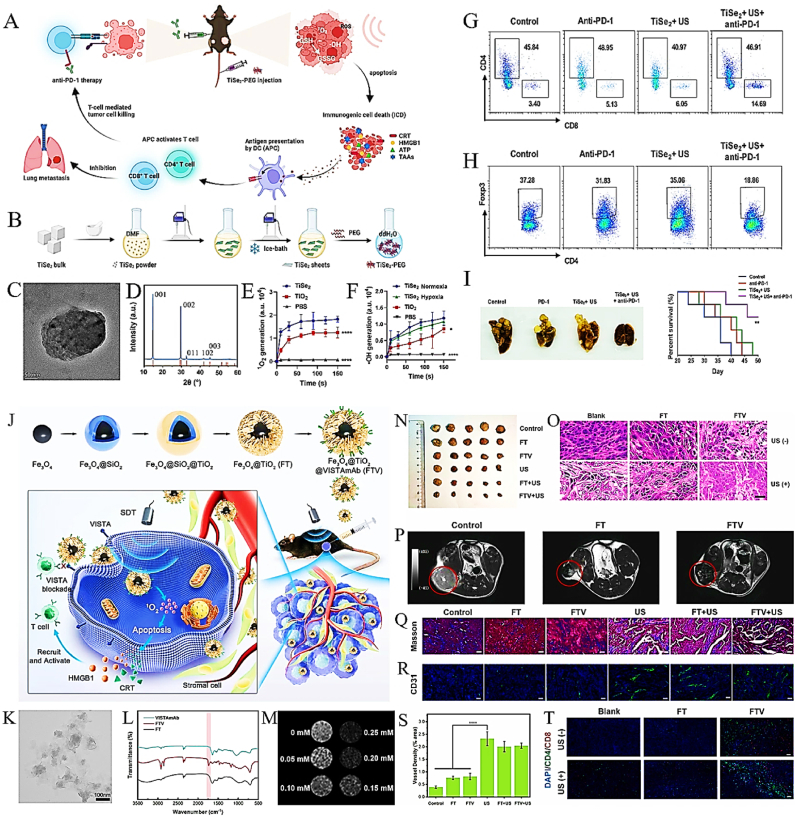
(A) Schematic representation of the synergistic integration between TiSe_2_-mediated SDT and anti-PD-1 checkpoint blockade in PDAC treatment. (B) The approach used to synthesize TiSe_2_ nanosheets. (C) High-resolution TEM images. (D) XRD spectra. (E) time-dependent generation of ^1^O_2_. (F) the time-dependent generation of ·OH generation. (G–H) Flow cytometric analysis delineating tumor-infiltrating CD8^+^ cytotoxic T lymphocytes (CD45^+^CD8^+^) and CD4^+^FoxP3^+^ regulatory T cells (CD45^+^CD4^+^FoxP3^+^) within distant tumor microenvironments. (I) The preparation process of Fe_3_O_4_@TiO_2_@VISTAmAb NPs and combining TiO_2_-based SDT with VISTA antibody treatment in PDAC. Adapted with permission from Ref. [30]. Copyright 2022 Springer Nature. (J) The preparation process of Fe_3_O_4_@TiO_2_@VISTAmAb NPs and combining TiO_2_-based SDT with VISTA antibody treatment in pancreatic cancer. (K) TEM images of FT NPs. (L) Micro-FTIR test of VISTAmAb, FT and FTV. (M) T2-weighted MR imaging of FTV under ultrasonic examination (1.0 MHz, 1.5 W cm^−2^, 50 % duty cycle) with different iron concentrations. (N) photographs of the tumor on day 14 after treatment. (O) H&E staining of tumor tissue after treatment. (P) T_2_-weighted MR imaging before and after the nanoparticle injection. Scale bar: 50 μm. Tumor tissue staining of different treatment groups (Q) Masson and (R) CD31. The analysis of CD31 (green color) in (S). (T) Immunofluorescence analysis of CD4 and CD8. Scale bar: 50 μm. Adapted with permission from Ref. [19]. Copyright 2024 Elsevier. (For interpretation of the references to color in this figure legend, the reader is referred to the Web version of this article.)

To address the dual challenges of hypoxia and immunosuppression in the PDAC, Chen et al. engineered stable titanium diselenide (TiSe_2_) nanosheets that synergistically integrate SDT with anti-PD-1 immune checkpoint blockade, achieving potent antitumor immunity in hypoxic microenvironments (Fig. 9A) [30]. The synthesis protocol, optimized for sonosensitizer functionality, produced TiSe_2_ nanosheets with lamellar morphology, as confirmed by TEM (Fig. 9B). Crystallographic analysis via XRD identified five distinct peaks, including (001), (002), and (003) facets, validating the hexagonal phase of TiSe_2_ (Fig. 9C). Functional characterization revealed time-dependent ^1^O_2_ generation under SDT conditions (Fig. 9D), with TiSe_2_ nanosheets exhibiting 2.3-fold higher ROS yields than conventional TiO_2_ nanoparticles. Notably, •OH production remained oxygen-independent, showing no significant difference between hypoxic (1 % O_2_) and normoxic (21 % O_2_) conditions (Fig. 9E), a critical advantage for treating PDAC's hypoxic core. In orthotopic PDAC models, the TiSe_2_ + anti-PD-1 combination therapy suppressed primary tumor growth by 68 % and attenuated metastatic dissemination (Fig. 9F). Mechanistically, this regimen elevated CD8^+^ T cell infiltration in distant tumors by 4.32-fold (14.69 % vs. 3.40 % in PBS controls) while maintaining stable CD4^+^ T cell populations (Fig. 9G), indicating selective activation of cytotoxic immunity. Concurrently, immunosuppressive Tregs were reduced by 49.40 % (18.86 % vs. 37.28 % in controls; Fig. 9H), reshaping the tumor immune landscape toward a pro-inflammatory phenotype. Strikingly, mice receiving combination therapy exhibited 63 % fewer metastatic lung nodules compared to monotherapy groups (Fig. 9I), demonstrating systemic antitumor efficacy. These findings establish TiSe_2_ nanosheets as a dual-functional platform that (1) overcomes hypoxia via oxygen-independent ROS generation and (2) synergizes with ICB to reverse immunosuppression. By bridging sonodynamic precision and immune reprogramming, this strategy addresses PDAC's therapeutic resistance at both metabolic and immunological levels, offering a transformative approach for recalcitrant hypoxic tumors.

Hong's group developed core-shell Fe_3_O_4_@TiO_2_@VISTAmAb (FTV) nanocomposites, synergizing magnetic resonance imaging (MRI)-guided SDT with VISTA-targeted immune checkpoint blockade (Fig. 9J) [19]. Structurally, FTV nanoparticles exhibited uniform spherical morphology with an average diameter of 120 nm (Fig. 9K), while Fourier-transform infrared spectroscopy (FTIR) confirmed successful VISTAmAb conjugation via a characteristic C–O stretching vibration peak at 1735 cm^−1^ (Fig. 9L). Functional characterization demonstrated concentration-dependent T_2_-weighted MRI signal attenuation, achieving a 2.8-fold contrast enhancement at 200 μg/mL (Fig. 9M), positioning FTV as a dual-modality theranostic agent. In orthotopic PDAC models, FTV + US treatment induced 72 % tumor weight reduction by day 14 compared to controls (Fig. 9N), with histopathological analysis revealing extensive nuclear pyknosis and karyolysis (Fig. 9O). MRI corroborated these findings, showing sharp tumor margin delineation via T_2_ dark signals (Fig. 9P), enabling real-time treatment monitoring. Mechanistically, US-triggered SDT disrupted the fibrotic stroma, reducing collagen density by 41 % (Fig. 9Q), while simultaneously enhancing tumor vascularization (Fig. 9R–S). Immunologically, FTV + US treatment elevated intratumoral CD8^+^ and CD4^+^ T cell infiltration by 3.2- and 1.8-fold, respectively (Fig. 9T), indicating reversal of the "immune-cold" phenotype. These results establish FTV nanocomposites as a triple-functional platform that: (i) Enables MRI-guided SDT for precision tumor targeting, (ii) Remodels fibrotic stroma to enhance drug/immune cell penetration, (iii) Blocks VISTA-mediated immunosuppression to amplify antitumor immunity. By integrating diagnostic imaging, stromal modulation, and immune reprogramming, this strategy addresses PDAC's therapeutic bottlenecks, offering a paradigm shift in precision oncology.

### 4.2 Synergistic mechanisms of SDT and ICB in PDAC

**Fig. 10:**
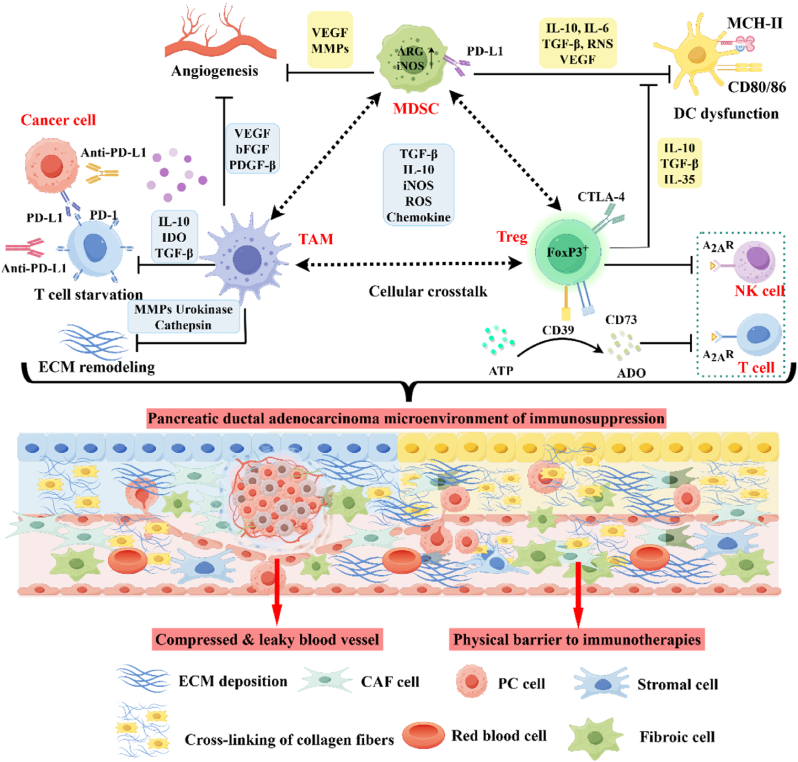
This schematic delineates the TME architecture comprising neoplastic populations, stromal constituents, and immunomodulatory elements, with particular focus on three dominant immunosuppressive cell lineages - TAMs, MDSCs, and Tregs - and their coordinated interplay in establishing immune-evasive niches through bidirectional cellular crosstalk. By Figdraw.

The clinical evidence from the emerging synergistic treatment of PDAC through sonodynamic and immune checkpoint modulation indicates that modulating the tumor microenvironment is crucial for enhancing the therapeutic effect. PDAC is a highly aggressive malignancy characterized by a dense desmoplastic stroma, hypoxic TME, and profound immunosuppression. Within the TME, tumor-associated macrophages (TAMs) as the predominant immune population demonstrate progressive upregulation of PD-1/PD-L1 axis activation during tumorigenesis, concurrently driving T cell functional exhaustion and immune checkpoint therapeutic resistance [[154], [155], [156]]. Myeloid-derived suppressor cells (MDSCs) comprise functionally heterogeneous subsets that orchestrate immunosuppressive networks, while Tregs serve as principal immunoregulators critically suppressing antitumor responses through immune homeostasis mogdulation (Fig. 10) [157,158]. The integration of SDT with ICB represents an innovative approach to overcome therapeutic resistance via mechanistically distinct pathways. SDT employs ultrasound-responsive agents (e.g., TiO_2_ nanoparticles, porphyrin derivatives) to spatially confine ROS generation, inducing direct tumor cytotoxicity while concurrently reprogramming the tumor immune landscape to enhance anti-PD-1/PD-L1 therapeutic responses. Therefore, this review systematically elucidates the underlying synergistic mechanisms by integrating evidence from preclinical models and emerging clinical trial data, with the aim of providing actionable insights to guide and inspire future clinical investigations.

#### 4.2.1 ROS induce immunogenic cell death

Sonodynamic therapy elicits immunogenic cell death in PDAC through ultrasound-activated sonosensitizers, triggering cytotoxic ROS generation to selectively eradicate tumor cells. The ICD process manifests as sequential release of damage-associated molecular patterns (DAMPs) — calreticulin (CRT) exposure, ATP secretion, and HMGB1 liberation — creating an immunostimulatory microenvironment that facilitates dendritic cell activation and subsequent adaptive immune responses [159,160]. CRT undergoes surface translocation to function as an immunogenic "eat-me" signal through LDL receptor-related protein 1 (LRP1) receptor engagement on dendritic cells. This molecular interaction initiates coordinated phagocytic clearance of tumor-derived material coupled with efficient antigen presentation machinery activation [[161], [162], [163], [164]]. Extracellular ATP activates P2X7 receptors on dendritic cells, initiating a cascade of NLRP3 inflammasome-mediated IL-1β release. This cytokine microenvironment drives CD8^+^ T-cell differentiation into Th1 effector lymphocytes, establishing tumoricidal immunity through interferon-γ-dependent mechanisms [165,166]. HMGB1, released during late-stage apoptosis, binds TLR4 on DCs, amplifying NF-κB signaling and upregulating co-stimulatory molecules (CD80/CD86) essential for T-cell activation. In PDAC models, SDT increased MHC-I expression on tumor cells, facilitating cross-presentation of neoantigens and resulting in a surge in tumor-infiltrating CD8^+^ T cells [30,167]. This ICD-driven immune priming establishes a reservoir of tumor-reactive T cells, but their activity is often suppressed by PDAC's immunosuppressive microenvironment. Critically, this sets the stage for ICB to reverse T-cell exhaustion and unleash adaptive immunity.

#### 4.2.2 ROS reprogram the immunosuppressive microenvironment

Building on the adaptive immune foundation laid by ICD, SDT-generated ROS act as molecular sculptors to remodel PDAC's immunosuppressive niche. The TME is predominantly infiltrated by immunosuppressive effector Tregs that constitutively express functional checkpoint molecules (CTLA-4, PD-1), costimulatory receptors (OX40), and chemotaxis-regulating surface proteins. These coordinated molecular signatures collectively establish immune-privileged niches through effector T-cell suppression and myeloid cell recruitment [168]. As the master transcriptional regulator of Treg development, FoxP3 orchestrates the coordinated upregulation of immunosuppressive effector molecules—including CD25 (IL-2Rα), CTLA-4, and GITR—through chromatin remodeling at locus control regions, thereby establishing the functional integrity of Treg-mediated immune tolerance [169,170]. ROS selectively deplete Tregs by oxidizing the transcription factor FoxP3, destabilizing their immunosuppressive identity and inducing apoptosis. Single-cell RNA sequencing in PDAC patient-derived xenografts (PDX) revealed a reduction in FOXP3^+^ Tregs post-SDT, accompanied by diminished TGF-β and IL-10 levels, key drivers of immune suppression [171]. Concurrently, ROS disrupt MDSC functionality through arginase-1 downregulation, effectively dismantling their T-cell inhibitory machinery. Significantly, these immunoregulatory cells exacerbate therapeutic resistance to ICB via dual mechanisms: fostering immunosuppressive niche formation through regulatory T-cell and cancer-associated fibroblast recruitment, while concurrently executing direct lymphocyte suppression [158]. In PDAC models, SDT reduced granulocytic MDSCs (G-MDSCs), restoring CD8^+^ T-cell proliferation [172]. ROS also repolarize TAMs from immunosuppressive M2 (CD206+/IL-10+) to pro-inflammatory M1 (iNOS+/IL-12+) phenotypes via STAT1-IRF5 activation, increasing CXCL9 production to recruit cytotoxic T lymphocytes [173,174]. Paradoxically, ROS upregulate PD-L1 on surviving tumor cells through NF-κB and HIF-1α pathways, creating a therapeutic vulnerability for anti-PD-L1 antibodies [[175], [176], [177]]. This dual role of ROS—depleting immunosuppressive cells while sensitizing tumors to ICB—transforms the TME from a barrier into a facilitator of antitumor immunity.

#### 4.2.3 Ultrasound enhances vascular and immune accessibility

**Fig. 11:**
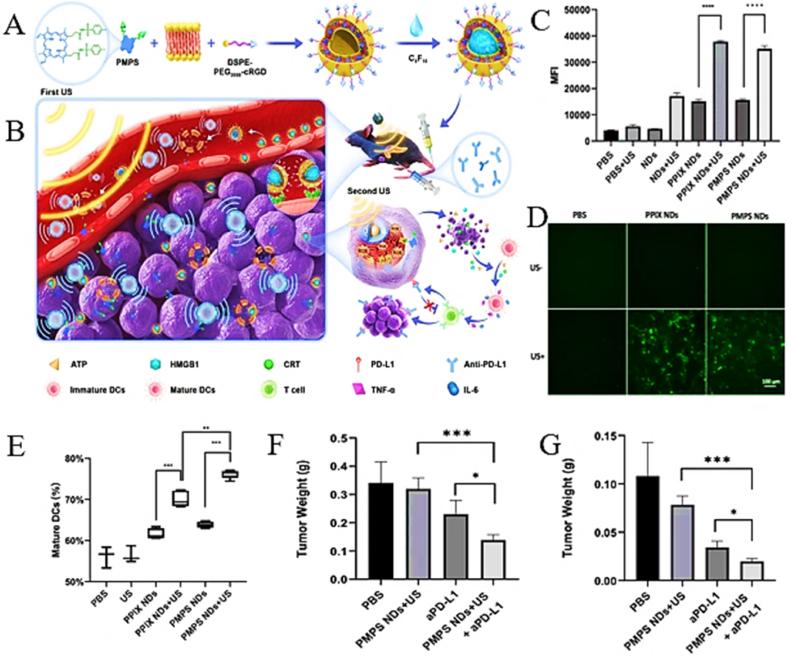
(A) Schematic illustration of the synthesis route of PMPS NDs. (B) The in vivo cavitation-assisted ER targeted SDT and aPD-L1 immunotherapy at primary tumor and distant metastasis tumor models. The sonodynamic effect of synthesized NDs (C) and cellular ROS generation (D) under US irradiation. (E) The DCs stained under different treatment and the quantitative data. The digital photo of orthotopic tumor (F) and the weight of distant tumor (G). Adapted with permission from Ref. [21]. Copyright 2022 Springer Nature.

The mechanical effects of ultrasound, particularly acoustic cavitation, synergize with ROS-driven molecular changes to overcome PDAC's physical barriers. Acoustic cavitation generates shear forces that transiently disrupt the dense desmoplastic stroma, reducing interstitial fluid pressure and decompressing collapsed blood vessels. For instance, Chen's team developed cavitation-enhanced endoplasmic reticulum-targeted nanodroplets (PMPS NDs, ∼329 nm). This innovative platform synergistically combines three therapeutic modalities: deep-tissue penetration through acoustic cavitation, organelle-specific SDT, and augmented anti-PD-L1 immunotherapy efficacy (Fig. 11A–B) [21]. Sonodynamic performance evaluation demonstrated superior ^1^O_2_ generation under US irradiation, with porphyrin fluorescence intensity surpassing non-targeted controls by 3.1-fold (Fig. 11C–D). This organelle-specific intervention induced marked ICD, as evidenced by 2.7-fold elevation in BMDC maturation rates (Fig. 11E). Therapeutic evaluation in PDAC models showed synergistic effects: combination therapy (PMPS NDs + US + aPD-L1) achieved 68 % reduction in primary tumor mass and 55 % suppression of metastatic growth (Fig. 11F–G), significantly outperforming individual treatment modalities. This triple-action platform demonstrates: (i) Stromal barrier penetration via cavitation physics; (ii) ER-targeted ICD induction for systemic immune activation; (iii) Immune checkpoint blockade potentiation. Concurrently, SDT promotes vascular normalization by downregulating VEGF and angiopoietin-2 secretion from CAFs, restoring endothelial cell integrity and perfusion. Alleviating hypoxia reverses adenosine-mediated T-cell suppression and rejuvenates mitochondrial metabolism in cytotoxic T lymphocytes (CTLs), evidenced an increase in oxygen consumption rates (OCR) post-treatment [178,179]. These mechanical and biochemical effects are synergistic: stromal disruption enhances ICB delivery, while normalized vasculature sustains T-cell metabolic fitness, enabling prolonged antitumor activity. By dismantling stromal and vascular barriers, ultrasound ensures that both therapeutic agents and immune effectors reach their targets, bridging molecular mechanisms with physical delivery.

#### 4.2.4 Innate immune activation sustains antitumor responses

The therapeutic cascade culminates in SDT-induced activation of the cGAS-STING pathway, which bridges innate and adaptive immunity. Mitochondrial damage from ROS releases mitochondrial DNA (mtDNA) into the cytosol, where it binds cyclic GMP-AMP synthase (cGAS) to produce 2'3'-cGAMP, a potent STING agonist. STING activation triggers TBK1-IRF3 signaling and robust type I interferon (IFN-I) production, which potentiate dendritic cell immunocompetence while augmenting CD8^+^ T lymphocyte cytotoxicity through MHC-I/II antigen presentation optimization [180,181]. IFN-I also upregulates PD-L1 on tumor cells via IRF1/STAT1, creating a feedforward loop that enhances ICB efficacy. Spatial transcriptomics in PDAC models revealed colocalization of STING-activated regions with PD-L1 + tumor cells and dense CD8^+^ T-cell clusters, illustrating the spatial coordination of these mechanisms [[182], [183], [184]]. In STING-deficient PDAC models, SDT-ICB combinations failed to control tumor growth, underscoring the pathway's indispensability. Conversely, STING agonists synergized with SDT to enhance IFN-β production and T-cell infiltration, suggesting combinatory potential. Thus, cGAS-STING signaling not only initiates innate immune responses but also sustains adaptive immunity, ensuring long-term tumor control in synergy with ICB.

### 4.3 The role of nanomedicine strategies in SDT-ICB synergy

The latest clinical case studies have further implicated the distinctive pancreatic tumor microenvironment in suboptimal therapeutic outcomes of SDT-ICB combination therapy, characterized by compromised drug delivery efficiency and diminished treatment efficacy. Consequently, the rational design of nanoparticles requires strategic engineering to (1) penetrate the stromal barrier, (2) achieve tumor cell-specific targeting, and (3) enable controlled drug release under harsh physicochemical conditions, thereby ensuring deep tumor penetration during SDT-ICB combination therapy.

#### 4.3.1 The role of Nanomedicine's physicochemical properties in SDT-ICB synergy

Owing to the physiological barriers that exist in PDAC, the process of nanoparticle penetration into the tumor must be considered when designing formulations. The size and morphology of NPs are determining physical factors for their biodistribution, tumor accumulation, cellular internalization, and physical energy conversion efficiency. Solid tumor tissues exhibit an enhanced permeability and retention (EPR) effect, yet their irregular vasculature and high interstitial fluid pressure constitute significant diffusion barriers [185,186]. Size directly influences the ability of NPs to penetrate these barriers. It is widely acknowledged that NPs within the 10–100 nm size range achieve an optimal balance between circulation time and tumor penetration depth [187,188]. While smaller NPs (<5 nm) can deeply penetrate tumors, they are rapidly cleared via renal glomerular filtration, resulting in short circulation half-lives and a tendency to diffuse back from tumor tissue into blood vessels, thereby reducing accumulation efficiency [189]. Conversely, excessively large NPs (>200 nm) are prone to capture and clearance by the mononuclear phagocyte system (MPS) in the liver and spleen, and struggle to extravasate from tumor vasculature or are confined to perivascular regions, failing to reach tumor cells distal to blood vessels [190,191]. In cancer SDT, when exposed to ultrasound irradiation, nanoparticles, serving as sonosensitizers, interact with substances such as H_2_O, OH^−^, and O_2_ in tumor tissues. This interaction triggers the production of ROS, ultimately destroying tumor cells. A decrease in particle size significantly increases the specific surface area nanoparticles, enhancing interaction between ultrasound-generated free electrons (e^−^) and holes (h^+^) and surrounding substances, elevating ROS generation [192]. The efficacy of SDT also relies on the uniform distribution of sonosensitizers at sufficiently high concentrations within the tumor. Size-optimized NPs achieve deeper penetration, ensuring that a broader range of sonosensitizers can be activated upon ultrasound irradiation, generating adequate levels of ROS and thermal effects, thereby eliminating deep-seated tumor cells and avoiding treatment blind spots [[55], [56], [57]]. Surface charge serves as the primary chemical factor governing the interactions between NPs and the biological microenvironment, dictating their fate at every stage from bloodstream circulation to ultimate cellular internalization [[193], [194], [195]]. The cell membrane exhibits a negative surface charge due to phospholipid headgroups and glycoproteins. As a result, positively charged NPs (cationic NPs) strongly adsorb to the membrane via electrostatic interactions, promoting cellular internalization [187]. However, they also bind non-specifically to non-targeted normal cells (e.g., red blood cells), leading to significant cytotoxicity and rapid opsonization by plasma proteins. This subsequently results in rapid clearance by the mononuclear phagocyte system (MPS), markedly shortening their bloodstream circulation time. In contrast, slightly negative or neutrally charged NPs, especially after PEGylation, minimize non-specific interactions with blood components. These “stealth” characteristics endow them with prolonged circulation half-lives, thereby enhancing their opportunity to accumulate in tumor tissues through the EPR effect [195,196]. TAMs are the most abundant immune cells within the TME and are typically polarized toward the M2 phenotype (alternatively activated), which promotes tumor growth, angiogenesis, and immunosuppression. Reprogramming M2-type TAMs to the M1 phenotype (classically activated) enables them to exert anti-tumor and pro-inflammatory functions. Surface charge represents a powerful lever to modulate NP interactions with macrophages and influence their polarization [196]. Cationic NPs, owing to their strong affinity for negatively charged cell membranes, are more readily internalized by macrophages. This often triggers the activation of the inflammasome pathway and the release of pro-inflammatory cytokines (e.g., IL-1β, TNF-α), thereby favoring M1 polarization and facilitating targeted M2-to-M1 repolarization within the TME. M1 macrophages are efficient antigen-presenting cells (APCs) and can secrete chemokines to recruit T cells [197,198]. Converting immunosuppressive M2-TAMs to the M1 phenotype can fundamentally transform “cold” tumors into “hot” tumors, reverse the immunosuppressive microenvironment, and create a favorable context for ICB therapy, substantially potentiating its therapeutic efficacy.

**Fig. 12:**
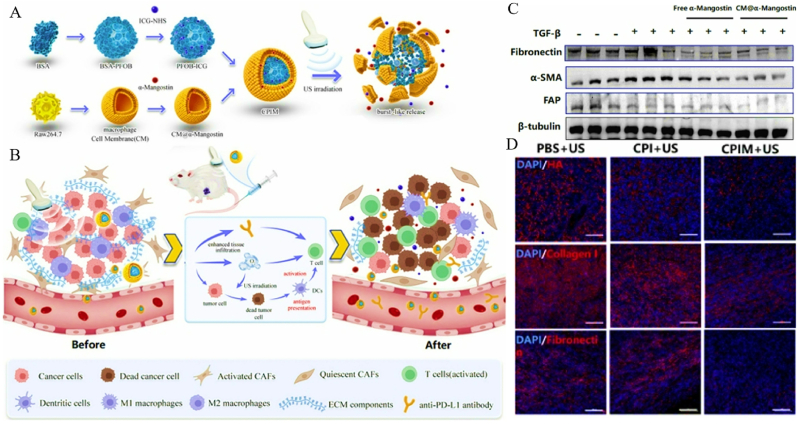
(A) A schematic diagram is presented to illustrate the CPIM structure and preparation strategy. (B) Schematic Illustration Mechanism of sonodynamic immunotherapy by regulating tumor stroma and reversing the immunosuppressive microenvironment of PDAC. (C) Western blot analysis of NIH-3T3 fibroblast lysates revealed dose-dependent modulation of fibrogenic markers (fibronectin, α-SMA, and FAP) following 72-h treatment with 20 μg/mL α-Mangostin or its chitosan-modified conjugate CM@α-Mangostin. Cellular responses were further characterized under fibrotic challenge conditions induced by 24-h pre-exposure to 10 ng/mL TGF-β before compound administration. (D) Immunofluorescence visualization of HA, Col I, and fibronectin deposition patterns in tumor stroma was systematically performed, with subsequent radiometric quantification of their respective MFI following combinatorial therapy employing ultrasound-activated nanoformulations. Adapted with permission from Ref. [207]. Copyright 2025 Springer Nature.

The targeted accumulation of nanomedicines within the tumor microenvironment is essential for achieving effective SDT-ICB combination therapy. The intratumoral biodistribution of therapeutic agents is governed by their physicochemical properties and the cellular phenotypes of tumors, with delivery limitations largely attributable to pathological alterations in the TME [199]. Specifically, the desmoplastic TME—characterized by abnormal vasculature, impaired lymphatic drainage, and chronic inflammation—elevates mechanical stress, induces hypoxia, and increases tumor interstitial fluid pressure (TIFP). These three barriers collectively hinder effective drug penetration [200]. In PDAC, the stroma is notably fibroinflammatory, enriched with ECM components such as hyaluronan, collagen, and proteoglycans, along with activated stromal cells [201]. This pathologically remodeled ECM further elevates TIFP and mechanical solid stress, generating considerable hydrodynamic resistance that limits drug perfusion [[202], [203], [204]]. To overcome these barriers, ECM degradation strategies have been developed, including the co-delivery of stromal-modulating enzymes. For instance, hyaluronidase-conjugated liposomes (PEGPH20) reduce hyaluronan content and decrease IFP in patient-derived xenograft (PDX) models, thereby improving the tumor-to-plasma ratio of gemcitabine [205]. Similarly, Luo et al. designed collagenase-loaded hollow titanium dioxide nanoparticles (Col@hTiO_2_ NPs), which combine matrix degradation with sonodynamic reactive oxygen species (ROS) generation. Under ultrasound irradiation, these particles triggered collagenase release and effectively degraded collagen fibers, reducing IFP and enhancing nanoparticle penetration and retention in PDX models through vascular normalization and pressure alleviation [206]. Furthermore, fibroblast-activation protein (FAP), highly expressed on cancer-associated fibroblasts (CAFs), represents a promising target for stromal modulation. Du et al. developed a macrophage membrane-coated system (CM@PFOB-ICG-α-Mangostin) for co-delivering oxygen and α-Mangostin via ultrasound-assisted extrusion [207]. The incorporated α-Mangostin suppresses CAFs and promotes stromal depletion, facilitating macromolecular infiltration into tumors (Fig. 12A and B). Western blot analysis confirmed that both free and nanocarrier-loaded α-Mangostin significantly reduced expression of fibronectin and α-SMA, indicating suppression of fibroblast activation (Fig. 12C). Masson staining further demonstrated that the combination of perfluorocarbon-loaded nanoliposomes and ultrasound reduced collagen I and hyaluronan expression (Fig. 12D). Additionally, α-Mangostin treatment led to marked fibronectin reduction, confirming effective CAF inhibition. These stroma-targeting strategies collectively enhance the penetration and efficacy of SDT-ICB therapy by remodeling the physical and biological barriers of the TME, thereby promoting synergistic antitumor effects.

#### 4.3.2 The role of smart microenvironment-responsive drug delivery systems in SDT-ICB synergy

The presence of tumor stromal barriers significantly restricts the penetration of conventional chemotherapeutic agents, leading to heterogeneous drug distribution, limited therapeutic outcomes, and increased risk of systemic toxicity. Smart drug delivery systems designed to respond to tumor-specific microenvironmental signals—such as acidic pH (resulting from hypoxia and aerobic glycolysis) [208,209], overexpressed enzymes (e.g., matrix metalloproteinases, MMPs) [210,211], and elevated GSH levels [212,213]—enable precise and controlled release of therapeutic agents at the tumor site. This targeting strategy enhances localized drug accumulation through pathophysiology-responsive mechanisms while minimizing off-target effects.

In the context of SDT-ICB combination therapy, such stimuli-activated platforms capitalize on biochemical gradients within the tumor microenvironment (e.g., pH < 6.5 extracellularly and 5.0–6.0 in endo/lysosomes vs. pH 7.4 in normal tissue) to achieve spatiotemporal control over therapeutic release [214]. Specifically, Two distinct pH-responsive drug release mechanisms demonstrate therapeutic precision: (1) Chemical conjugation of therapeutic agents via acid-labile linkages (phosphoramidate [215], imine [216], orthoester [217], hydrazone bonds [[218], [219], [220]]) enables pH-dependent payload liberation through hydrolytic cleavage; (2) Smart nanovectors incorporating ionizable moieties undergo programmable structural transitions (solubility modulation/conformational switching) across physiological pH gradients [221]. Notably, hydrazone-based linkers offer programmable stability—remaining stable at physiological pH (7.4) yet rapidly hydrolyzing in acidic compartments (pH 4.0–6.0). Capitalizing on this mechanism, Ma et al. conjugated doxorubicin (DOX) to hydrophobic PVV via a hydrazone bond to achieve triggered release at pH ~ 5.0 [222]. The system exhibited enhanced structural stability, improved cellular uptake, and stronger anti-proliferative effects at tumor-mimetic pH (6.7), along with deeper penetration into tumor cores in ex vivo models. These smart release strategies potentiate SDT-ICB therapy by ensuring high local drug availability, promoting immunogenic sonodynamic effects, and enhancing immune cell infiltration through improved stromal penetration.

**Fig. 13:**
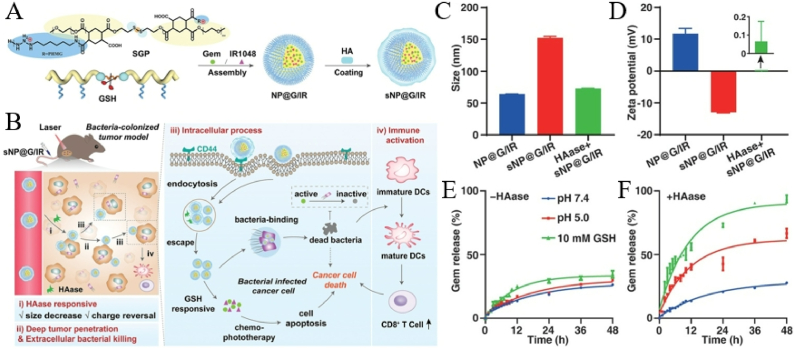
(A) Preparation process of the dual-cascade responsive sNP@G/IR with core–shell structure. (B) sNP@G/IR suppresses bacteria-colonized tumor growth by eliminating tumor-resident intracellular bacteria and precise drug delivery. (C) Particle size and (D) zeta potential of NP@G/IR, sNP@G/IR, and sNP@G/IR incubated with HAase. Gem release profiles of sNP@G/IR in different conditions: E) without HAase and F) with Haase. Adapted with permission from Ref. [213]. Copyright 2022 Wiley.

Stimulus-responsive drug delivery systems utilizing ROS-cleavable thioketal linkages or GSH-sensitive disulfide bridges have been widely demonstrated to enable precise therapeutic release under pathological conditions. In HeLa cell studies, dual-responsive systems integrating both GSH-triggered disulfide bonds and ROS-activated mechanisms showed significantly higher intracellular drug accumulation after 4 h and 12 h compared to single-response systems (*P* < 0.005) [212], attributable to synergistic amplification of tumor-specific release pathways. These mechanisms are particularly advantageous for SDT-ICB combination therapy, as SDT generates substantial ROS in situ, which can be harnessed for spatiotemporally controlled drug liberation, thereby enhancing immunogenic cell death and potentiating immune checkpoint blockade. This enhanced performance stems from the synergistic amplification of tumor-specific drug release mechanisms. Moreover, externally triggered modalities like near-infrared (NIR)-absorbing gold nanocages enable precise photothermal drug release, with 808 nm laser irradiation (1 W/cm^2^) inducing 80 % payload release within 5 min [223]. For example, Kang et al. reported a dual-cascade responsive nanoparticle (sNP@G/IR) that can sequentially trigger deep penetration [213]. sNP@G/IR consists of a HA shell and GSH-responsive polymer-core (NP@G/IR), that encapsulates gemcitabine (Gem) and photothermal agent (IR1048) (Fig. 13A). sNP@G/IR actively homes in on the tumor due to the CD44 targeting of the HA shell, which is subsequently degraded by the hyaluronidase in the extracellular matrix (Fig. 13B). The engineered NP@G/IR nanoparticles exhibited optimized tumor penetration characteristics through size reduction (hydrodynamic diameter: 73.0 nm) and surface charge reversal (zeta potential: +0.1 mV), which were triggered by hyaluronidase-mediated degradation of the HA shell in sNP@G/IR (Fig. 13C–D). Afterward, Gem release profiles were evaluated in different solutions (Fig. 13E–F). The redox-responsive drug release profile demonstrated concentration-dependent characteristics: under 10 mM GSH, sNP@G/IR achieved 34.55 % Gemcitabine liberation over 48 h. Following HAase-mediated hyaluronic acid shell removal, NP@G/IR maintained structural stability at physiological pH (7.4), whereas reductive microenvironment simulation triggered rapid payload discharge (93.75 % within 4 h). This dual-responsive design supports prolonged circulation and tumor-specific drug activation, making it highly suitable for enhancing SDT-ICB therapy through improved penetration and controlled release of both sonosensitizers and immunomodulators.

#### 4.3.3 The role of mechanical force-driven in SDT-ICB synergy

**Fig. 14:**
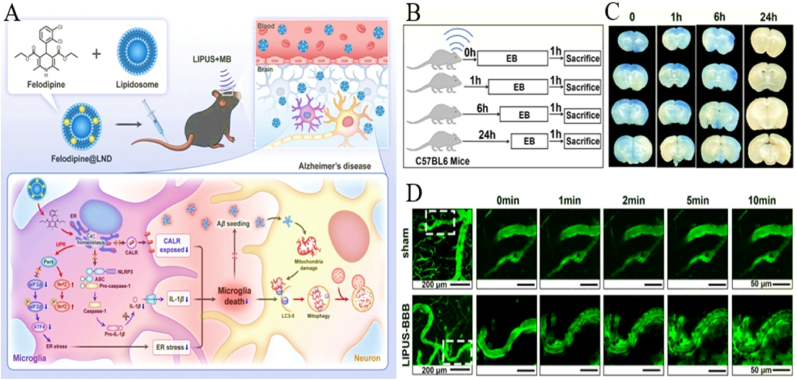
(A) Preparation and LIPUS – assisted delivery of felodipine nanodrug (felodipine@LND). **(**B) Experimental design for verification for LIPUS - BBB opening. **(**C) Evans blue staining at 0 h, 1 h, 6 h and 24 h after LIPUS. **(**D) Two-photon imaging and comparison analysis for coumarin 6@LNPs permeation in brain parenchyma at different times after tail intravenous injection of coumarin 6@LNPs between sham and LIPUS-BBB groups. Adapted with permission from Ref. [231]. Copyright 2024 Wiley. (For interpretation of the references to color in this figure legend, the reader is referred to the Web version of this article.)

Mechanical forces—including focused ultrasound, thermal energy, microneedle arrays, and magnetic fields—have been strategically employed to alleviate solid stress within the tumor microenvironment. These approaches act synergistically with nanotherapeutic systems to significantly improve intratumoral drug delivery efficiency [[224], [225], [226], [227]]. Among these, ultrasound-mediated strategies offer particularly high compatibility with SDT-ICB combination therapy by leveraging both biological and physical mechanisms. The integration of ultrasound with nanoparticle delivery systems produces multi-level anticancer synergies. Through thermal and mechanical effects, ultrasound enhances the extracellular transport of drugs and nanoparticles. Ultrasonic thermal activation (40–43 °C) facilitates tumoral drug delivery via three key mechanisms: Mild hyperthermia augments vascular perfusion and endothelial permeability, promoting nanoparticle extravasation into tumor tissue; Microbubble-induced cavitation generates localized shear forces and microstreaming, concurrently triggering payload release and transiently disrupting vascular barriers; Acoustic radiation forces promote perivascular nanoparticle accumulation with deeper penetration than passive diffusion [228]. Notably, ultrasound-triggered sonopermeation has been clinically applied for non-invasive modulation of biological barriers, such as the blood-brain barrier (BBB), demonstrating enhanced therapeutic delivery in human trials [229,230]. For instance, Lammers' team developed an ultrasound-responsive liposomal system (felodipine@LND) for Alzheimer's therapy, using low-intensity pulsed ultrasound (LIPUS) to achieve targeted hippocampal drug delivery (Fig. 14A) [231]. Evans blue staining confirmed BBB opening immediately after sonication, with recovery within 24 h (Fig. 14B and C). Two-photon imaging further showed increasing accumulation of Coumarin 6-labeled nanoparticles in brain parenchyma within 10 min post-injection (Fig. 14D). Building on this, Sulheim et al. elucidated a dual mechanism: sonopermeation simultaneously modulates vascular integrity and remodels extracellular matrix architecture, synergistically enhancing interstitial drug penetration [232]. These insights are highly relevant to SDT-ICB therapy, where ultrasound can not sonosensitizer activation and immunostimulatory cascades. By mitigating stromal barriers, ultrasound significantly enhances the delivery and efficacy of both sonodynamic and immunotherapeutic agents, underscoring its critical role in synergistic cancer treatment.

## 5 Conclusions and future perspectives

### 5.1 Conclusions

As metastatic spread and local tumor recurrence account for most cancer-related fatalities, targeting the tumor microenvironment's immunosuppressive nature to restore immune function presents a promising therapeutic avenue. SDT has emerged as a promising minimally invasive modality over the past decade, particularly for deep-seated malignancies like pancreatic ductal adenocarcinoma. Its clinical value derives from exceptional tissue penetration capabilities, enabling precise targeting of conventionally inaccessible lesions. While ICB demonstrates notable clinical efficacy, its utility is constrained by dose-limiting toxicities and acquired resistance. Common immune-related adverse events include hematologic toxicities (neutropenia, thrombocytopenia) and systemic manifestations (nausea, vomiting, fatigue), with alopecia being particularly prevalent [233]. More critically, the dual barriers of profound immunosuppression and low antigenicity in pancreatic TME fundamentally limit immunotherapy effectiveness. Some clinical studies have successfully addressed these biological challenges by integrating the strategies of SDT and ICB through complementary mechanisms: (i) SDT-induced immunogenic cell death reverses TME immunosuppression; (ii) ICB potentiates systemic antitumor immunity; (iii) Combined spatial and immune modulation establishes durable immunologic memory. This synergistic paradigm demonstrates enhanced metastatic control while mitigating relapse risks, representing a multidimensional solution to PDAC's therapeutic paradox. Finally, this review also pointed out the critical design principles for nanomedicines for SDT-assisted immunotherapy to enhance the therapeutic effect of PDAC, which had provided exciting opportunities for functional biomaterials in fighting against cancer.

### 5.2 Study limitations

While preclinical research has achieved notable breakthroughs, persistent knowledge gaps demand resolution to bridge the bench-to-bedside transition.

Firstly, early tumor detection remains pivotal for enhancing PDAC outcomes. The development of SDT-integrated theranostic platforms offers transformative potential by unifying diagnostic imaging and therapeutic intervention within a single system. This dual-capacity approach enables high-precision tumor mapping (morphology, localization), real-time visualization of therapeutic efficacy, and dynamic treatment protocol optimization. Through continuous feedback mechanisms, such systems facilitate adaptive treatment personalization, addressing tumor heterogeneity while minimizing procedural delays.

Secondly, the clinical translation of nanoscale sonodynamic agents necessitates comprehensive evaluation of their longitudinal biosafety profiles. While current preclinical evaluations predominantly rely on standardized toxicological assessments—including hematological screening, organ function indices, and acute biocompatibility metrics—these conventional paradigms primarily address short-term safety endpoints. Critical knowledge gaps persist regarding nanoplatforms' chronic metabolic fates and cumulative biological interactions. To bridge this translational disconnect, implementation of extended observation protocols tracking delayed physiological responses is strongly advised. Furthermore, biosafety validation across phylogenetically diverse animal models would better account for interspecies metabolic variability, particularly given the heterogeneity observed in human nanoparticle pharmacokinetics.

Third, current studies frequently omit justification for selected ultrasound irradiation parameters, while inconsistent energy delivery protocols hinder cross-comparison of sonosensitizer efficacy. Standardization of acoustic parameter optimization thus emerges as a critical requirement for reproducible therapeutic outcomes.

Fourth, the development of physiologically accurate tumor models remains crucial for evaluating SDT and other energy-based modalities. Current subcutaneous murine implantation paradigms fail to recapitulate human TME complexity, particularly compromising ultrasound's intrinsic penetration advantages and depth-dependent therapeutic assessment. These anatomical discrepancies between superficial animal models and deep-seated human malignancies hinder reliable efficacy evaluation while obscuring energy attenuation dynamics. Prioritizing orthotopic patient-derived xenograft systems that preserve native tumor heterogeneity and pathway interactions would significantly enhance preclinical-to-clinical translation.

Fifth, current administration methods for immune checkpoint inhibitors in SDT-ICB combination therapies (Table 2) predominantly employ systemic delivery routes, resulting in suboptimal tumor targeting and elevated off-target toxicity. Nanocarrier-mediated delivery systems enabling tumor-selective accumulation and stimuli-responsive release mechanisms should be prioritized in subsequent preclinical development. This strategic formulation approach promises enhanced therapeutic precision while mitigating systemic exposure risks.

Sixth, PDAC immunotherapy primarily targets the CTLA-4 and PD-1/PD-L1 axes to counteract tumor microenvironment immunosuppression. Clinical trials demonstrate superior safety and efficacy with dual checkpoint blockade compared to monotherapies, yet significant patient subgroups exhibit therapeutic resistance. This refractoriness likely stems from both unidentified immunoregulatory pathways and incomplete characterization of immune cell functional dynamics within malignant niches.

Seventh, the evolving spatiotemporal dynamics of tumor-immune-stromal crosstalk within the immune microenvironment present fundamental challenges for therapeutic interventions. Critical optimization requirements for SDT-ICB synergy involve dual preservation of checkpoint inhibitor integrity against sonosensitizer-generated oxidative stress, while ensuring spatiotemporally coordinated activation of both modalities to maximize tumor-suppressive synergy.

Eighth, scalable manufacturing protocols for engineered nanosensitizers remain pivotal for clinical translation, though current synthesis complexities and prohibitive production costs hinder industrial-scale implementation. While preclinical studies demonstrate promising therapeutic efficacy of novel nanoscale sonodynamic agents, their clinical adoption requires prioritized development of cost-effective fabrication methodologies to ensure therapeutic accessibility.

### 5.3 Prospects

Tumorigenesis fundamentally involves coordinated genetic and epigenetic aberrations that orchestrate malignant transformation across all cancer hallmarks. Emerging evidence highlights the therapeutic potential of epigenetic nanoregulators ("nano-epidrugs") targeting molecular modifications in DNA (5-methylcytosine) [234], RNA (N6-methyladenosine) [235], and protein (ubiquitination) hierarchies [236]. Integrating these epigenetic nano-formulations with immune checkpoint blockade demonstrates synergistic potential in overcoming therapeutic resistance and augmenting photodynamic efficacy across hematologic and solid malignancies [237]. Future development should prioritize rational engineering of sonosensitizers with epigenetic pathway modulation capabilities to optimize ICB-combination strategies. In conclusion, nanoparticle-mediated sonodynamic therapy exhibits a robust tumor-ablative effect while offering multiple advantages, including enhanced penetration depth and an improved safety profile. When synergized with immune checkpoint inhibitors (ICIs), this SDT/ICB combination effectively targets PDAC by inducing durable antitumor immunity and long-term immune memory, thereby preventing relapse. The limitations of monotherapy are addressed through synergistic mechanisms, achieving superior tumor eradication efficacy based on the principle of "synergistic interaction (CI < 1)". However, several challenges remain in the SDT/ICB modality, such as hypoxia-induced ROS resistance, suboptimal T-cell infiltration, and low permeability of nanomedicines. Future studies may address these issues by developing activatable nanocarriers or optimizing treatment protocols to expedite clinical translation.

## CRediT authorship contribution statement

**Haijie Li:** Writing – original draft, Software, Resources, Data curation, Conceptualization. **Ying Lei:** Supervision, Software. **Yunqing Tian:** Visualization. **Hang Nie:** Supervision, Conceptualization. **Yang Li:** Writing – review & editing. **Yu Mi:** Writing – review & editing, Writing – original draft, Supervision.

## Funding

This work was supported by the 10.13039/501100012166National Key R&D Program of China (2023YFA0914300), 10.13039/501100001809National Natural Science Foundation of China (22308278), and Shaanxi Province Key Research and Development Program (2025SFYBXM269).

## Declaration of competing interest

The authors declare that they have no known competing financial interests or personal relationships that could have appeared to influence the work reported in this paper.

## Data Availability

No data was used for the research described in the article.
